# Venomics and Peptidomics of Palearctic Vipers: A Clade-Wide Analysis of Seven Taxa of the Genera *Vipera*, *Montivipera*, *Macrovipera*, and *Daboia* across Türkiye

**DOI:** 10.1021/acs.jproteome.4c00171

**Published:** 2024-07-09

**Authors:** Maik Damm, Mert Karış, Daniel Petras, Ayse Nalbantsoy, Bayram Göçmen, Roderich D. Süssmuth

**Affiliations:** † Institut für Chemie, Technische Universität Berlin, Straße des 17. Juni 135, 10623 Berlin, Germany; ‡ LOEWE-Centre for Translational Biodiversity Genomics, Senckenberganlage 25, 60325 Frankfurt am Main, Germany; § Institute for Insect Biotechnology, Justus-Liebig University Giessen, Heinrich-Buff-Ring 26-32, 35392 Gießen, Germany; ∥ Program of Laboratory Technology, Department of Chemistry and Chemical Process Technologies, Acıgöl Vocational School of Technical Sciences, Nevşehir Hacı Bektaş Veli University, Acıgöl, 50140 Nevşehir, Türkiye; ⊥ Department of Biochemistry, University of California Riverside, 169 Aberdeen Dr, Riverside, California 92507, United States; # Interfaculty Institute of Microbiology and Infection Medicine, University of Tuebingen, Auf der Morgenstelle 24, 72076 Tuebingen, Germany; ¶ Department of Bioengineering, Faculty of Engineering, Ege University, Bornova, 35100 Izmir, Türkiye; ∇ Zoology Section, Department of Biology, Faculty of Science, Ege University, Bornova, 35100 Izmir, Türkiye

**Keywords:** venom, snakebite, proteomics, peptidomics, viper

## Abstract

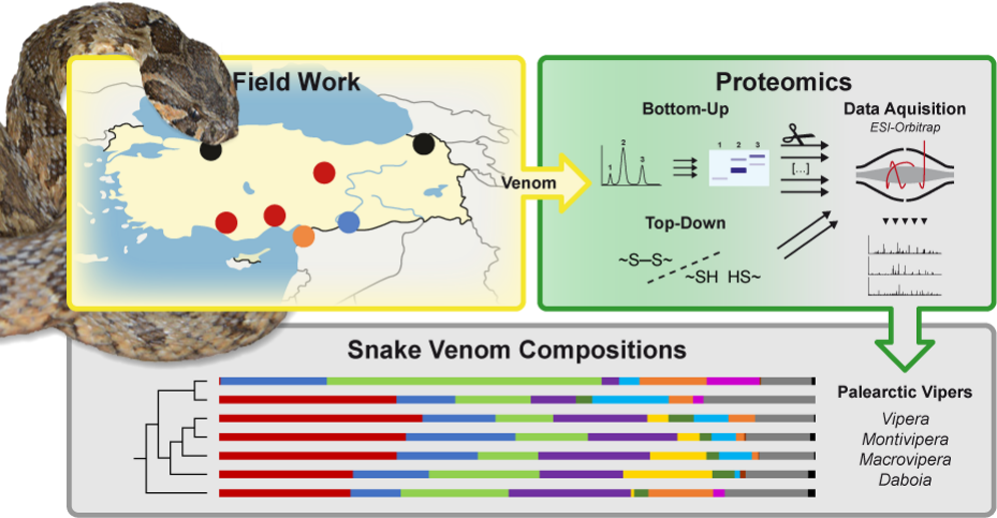

Snake venom variations are a crucial factor to understand the consequences of snakebite envenoming worldwide, and therefore it is important to know about toxin composition alterations between taxa. Palearctic vipers of the genera *Vipera*, *Montivipera*, *Macrovipera,* and *Daboia* have high medical impacts across the Old World. One hotspot for their occurrence and diversity is Türkiye, located on the border between continents, but many of their venoms remain still understudied. Here, we present the venom compositions of seven Turkish viper taxa. By complementary mass spectrometry-based bottom-up and top-down workflows, the venom profiles were investigated on proteomics and peptidomics level. This study includes the first venom descriptions of *Vipera berus barani*, *Vipera darevskii*, *Montivipera bulgardaghica albizona,* and *Montivipera xanthina*, as well as the first snake venomics profiles of Turkish *Macrovipera lebetinus obtusa*, and *Daboia palaestinae*, including an in-depth reanalysis of *M. bulgardaghica bulgardaghica* venom. Additionally, we identified the modular consensus sequence pEXW(PZ)_1–2_P(EI)/(KV)PPLE for bradykinin-potentiating peptides in viper venoms. For better insights into variations and potential impacts of medical significance, the venoms were compared against other Palearctic viper proteomes, including the first genus-wide *Montivipera* venom comparison. This will help the risk assessment of snakebite envenoming by these vipers and aid in predicting the venoms’ pathophysiology and clinical treatments.

## 1 Introduction

Snakebite envenoming is a major burden on global health.^1−3^ More than 5.4 million annual snakebites cause more than 150,000 casualties and several more long-lasting physical as well as often neglected mental disabilities.^4−7^ Responsible for a high number of these snake encounters are, beside elapids (Elapidae) and pit vipers (Crotalinae), the “true” or Old World vipers (Viperinae).^8^ Several taxa within this subfamily are in the focus of epidemiological snakebite envenoming dynamics and venom research.^9−14^ Among them, are the particularly relevant Palearctic vipers of the genera: *Vipera*, *Montivipera*, *Macrovipera* and *Daboia*. They consist of about 35 species, but their taxonomic classification has been a topic of debate for long time.^15−17^ The World Health Organization WHO lists all four genera at the highest medical importance, Category 1, with strong impact across their distributions.^8,10,18−21^

**Figure 1:**
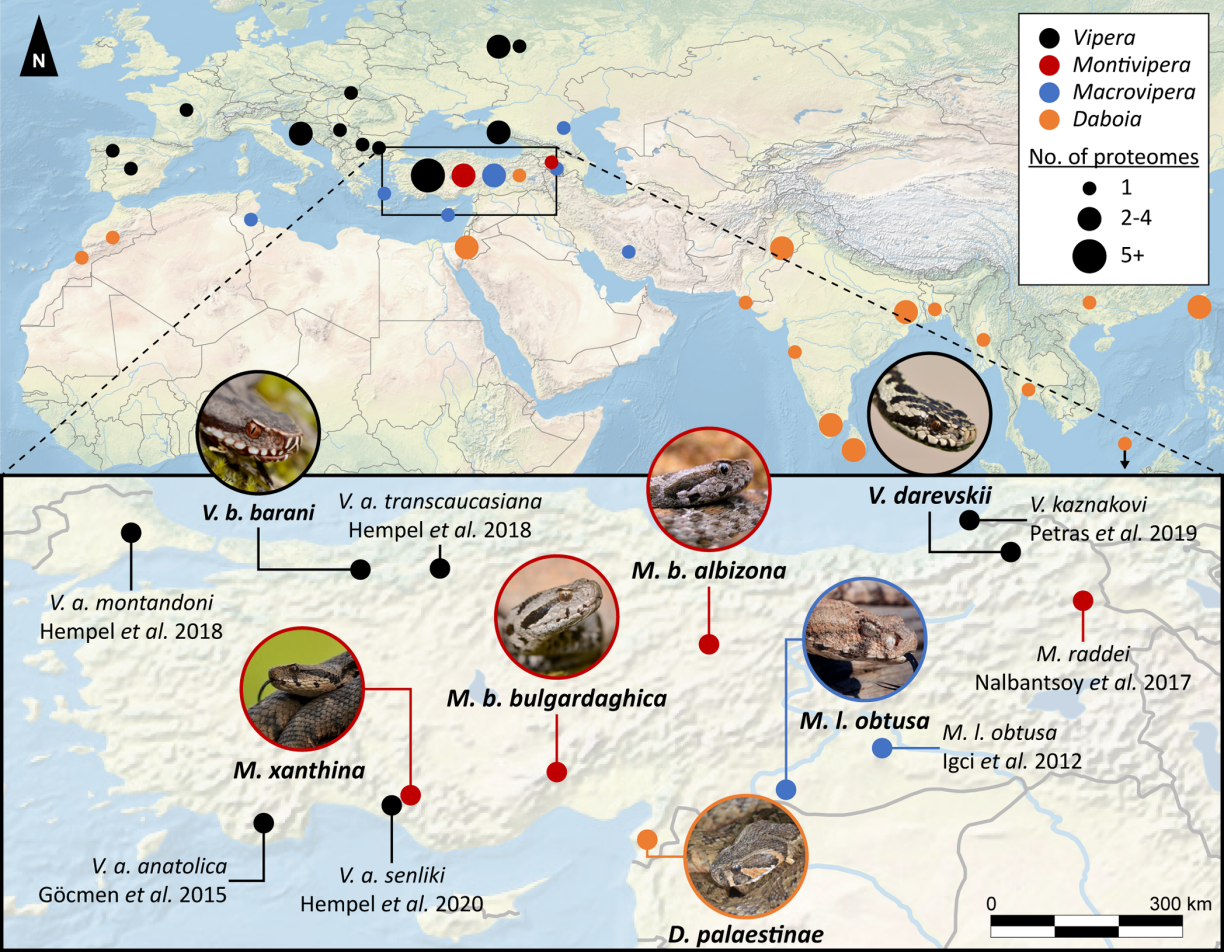
Mapped venomics studies of four Palearctic viper genera from 2003 to 2023. *Vipera* (black), *Montivipera* (red), *Macrovipera* (blue), and *Daboia* (orange) from different geographical areas within 2003 to 2023. The bottom map shows the zoomed detailed overview of venomics studies on Turkish viper taxa with the original studies. Investigated taxa in this study are shown by images of the corresponding snake. Samples/specimen of nonreported venom origin were allocated to the respective capital city of the country. Closely located samples were summed to disks of increasing size. All snake images by Bayram Göçmen, except *Daboia* by Mert Karış.

Viper envenomation are characterized by mostly hemotoxic and tissue damaging clinical effects, while neurotoxic effects are more uncommon.^22−25^ Responsible for this spectrum of symptoms are more than 50 known toxin families in snake venoms, which are often functionally modulated via posttranslational modifications.^26−28^ Viperine venoms are primarily composed by enzymatic (e.g., proteases, lipases, oxidases) and nonenzymatic (e.g., lectins, growth factors, hormones) components extending molecular sizes across four magnitudes from small peptides of <500 Da up to protein complexes of >120 kDa.^29,30^ Over the past decade, venoms of Palearctic vipers have been intensively analyzed on the proteomic level for 20 species across 25 countries (Figure 1).

Remarkably, a large number of species and most subspecies have never been analyzed by state of the art approaches, like modern venomics.^13,31^ Investigating these neglected taxa will help to predict the effect of a snakebite envenoming, to optimize treatment strategies, but also unveil venom evolutionary ecology and guide biodiscovery.^28,32−36^ Especially the proteomic bottom-up (BU) “snake venomics” approach, a three-step protocol with a final HPLC (high performance liquid chromatography) linked high resolution mass spectrometry (MS) peptide detection, gives insights into compositions and allows cross-study comparison.^37−39^ Therefore, it has been used to correlate snake venoms in larger biogeographic contexts.^13,40−42^

On the border between Europe and Asia, Türkiye represents a hotspot of snake diversity, hosting members of all four Palearctic viper genera.^15,43−45^ Similar to tropical and subtropical regions, snakebite represents a major health burden in Türkiye, but the exact magnitude remains unclear due to the lack of comprehensive data.^46−48^ Only a few studies address concrete numbers about snakebite envenoming in Türkiye.^46,49,50^ While awareness of snakebite grows, the species responsible for a bite are often not known. It is therefore necessary to investigate the range of venomous snakes in the country and the extent to which their venoms are composed. In the past decade, a few of these Turkish species have been studied using modern venomics approaches (Figure 1). These include a few representatives of Viperinae (*Vipera*, *Montivipera,* and *Macrovipera*), as well as Morgan’s desert cobra, *Walterinnesia morgani* as the only elapid within this region.^13,51−57^ Therefore, venom composition and potentially unfolding effects of envenoming stemming from their components are largely unknown hindering therapeutically care of snakebite victims.

Here, we set out to fill this knowledge gap and investigate the venom composition of seven Turkish viper taxa, many of which being recognized as threats to health.^18^ Specifically, we investigate representatives of each Turkish viperine genus by a combination of BU snake venomics and top-down (TD) proteomics including peptidomics.^58,59^ We describe for the first time the venom composition of the Baran’s adder *Viperaberus barani* (Böhme and Joger, 1983), an endemic subspecies of the adder located on the north of Türkiye, and the Darevsky’s viper *Viperadarevskii* (Vedmederja et al., 1986), a small critically endangered viper living in close proximity to the Turkish-Georgian-Armenian border.^60,61^ Furthermore, aiming to gain a deeper understanding of the mountain viper venoms, we provide insights into the closely related *Montiviperaxanthina* complex: *Montiviperabulgardaghica bulgardaghica* (Nilson and Andren, 1985) and *M. bulgardaghica albizona* (Nilson et al., 1990), as well as the Ottoman Viper *M. xanthina* (Gray, 1849).^43,45,62−64^ The other two genera are represented by one blunt-nosed viper subspecies *Macrovipera lebetinus obtusa* (Dwigubsky, 1832) and the most northern, newly described Anatolian specimen of the Palestine viper *Daboia palaestinae* (Werner, 1938).^65−67^

By extensive modern venomics analysis we double the number of reported Turkish vipers venom compositions and gain novel insights in the venom variation of the four Old World viper genera *Vipera*, *Montivipera*, *Macrovipera,* and *Daboia* on the proteomics as well as peptidomics level.

## 2 Materials and Methods

### 2.1 Origin of Snake Venoms

All snakes were wild caught within Türkiye, the collections were approved with ethical permissions (Ege University, Animal Experiments Ethics Committee, 2010–2015) and special permissions (2011–2015) for field studies from the Republic of Türkiye, Ministry of Forestry and Water Affairs were received. For a detailed list of permission numbers, locations of collection and further venom pool information, see Supporting Information Table S1.

### 2.2 Bottom-Up Proteomics—Snake Venomics

The used bottom-up protocol is adapted from published protocols.^56,68^ In short, 1 mg lyophilized venom was fractionated by HPLC, observed at 214 nm. Collected peaks were submitted to SDS-PAGE profiling and in-gel tryptic digestion, followed by LC–MS/MS measurements. The detailed protocol steps are placed in the Supporting Information under Additional Materials and Methods (Detailed Bottom-up proteomics—Snake Venomics).

For the MS analysis, the extracted and dried tryptic peptides were redissolved in 30 μL aqueous 3% (*v*/*v*) ACN with 1% (*v*/*v*) HFo, and 20 μL of each was injected into an LTQ Orbitrap XL mass spectrometer (Thermo, Bremen, Germany) via an Agilent 1260 HPLC system (Agilent Technologies, Waldbronn, Germany) using a reversed-phase Grace Vydac 218MS C18 (2.1 × 150 mm; 5 μm particle size) column. The detailed LC–MS parameters and Bottom-up data analysis workflow are placed in the Supporting Information under Additional Materials and Methods (Detailed Bottom-up proteomics—Mass Spectrometry).

### 2.3 Bottom-Up Data Analysis

The BU LC–MS/MS data RAW files were converted into the MASCOT generic file format (MGF) using MSConvert (version 3.0.10577 64-bit) with peak picking (vendor msLevel = 1−).^69^ For an automated database comparison, files were analyzed using pFind Studio,^70^ with pFind (version 3.1.5) and the integrated pBuild, with the following parameters: MS Data (format: MGF; MS instrument: CID-FTMS); identification with Database search (enzyme: Trypsin KR_C, full specific up to 3 missed cleavages; precursor tolerance +20 ppm; fragment tolerance +20 ppm); open search setup with fixed carbamidomethyl [C] and Result Filter (show spectra with FDR ≤ 1%, peptide mass 500–10,000 Da, peptide length 5–100 amino acids, and show proteins with number of peptides >1 and FDR ≤ 1%). The used databases included UniProt “Serpentes” (ID 8750, reviewed, canonical and isoform, 2640 entries, last accessed on 8th April 2021 via https://www.uniprot.org/) and the Common Repository of Adventitious Proteins (215 entries, last accessed on 10th February 2022; available at https://www.thegpm.org/crap/index.html). The results were batch-exported as PSM score of all peptides identified with pBuild and manually cleared from decoy entries, contaminations, and artifacts to generate the final list of unique peptide sequences per sample with the best final score. For a second confirmation of identified sequences, all unique entries were analyzed using BLAST search with blastp against the nonredundant protein sequences (nr) of the “Serpentes” (taxid: 8570) database.^71,72^ In case of nonautomatically annotated band identity, files were manually checked using Thermo Xcalibur Qual Browser (version 2.2 SP1.4), de novo annotated, and/or compared on MS1 and MS2 levels with other bands to confirm band and peptide identities. Deconvolution of isotopically resolved spectra was carried out by using the XTRACT algorithm of Thermo Xcalibur.

### 2.4 Top-Down Proteomics

The used top-down protocol is adapted from published protocols.^54,68^ In short, 100 μg lyophilized venom was measured reduced and nonreduced. Ten μL of each sample was injected into an Q Exactive HF mass spectrometer (Thermo, Bremen, Germany) via a Vanquish ultrahigh performance liquid chromatography (UHPLC) system (Agilent Technologies, Waldbronn, Germany) using a reversed-phase Supelco Discovery BIO wide C18 (2.0 × 150 mm; 3 μm particle size; 300 Å pore size) column thermostated at 30 °C. The detailed protocol steps are placed in the ○ under Additional Materials and Methods (Detailed Top-down proteomics—Mass Spectrometry).

### 2.5 Top-Down Data Analysis

The TD LC–MS/MS Thermo RAW data were converted to a centroided MS data format (mzML) using MSConvert (version 3.0.10577 64-bit) with peak picking (vendor msLevel = 1−) and further analyses by TopPIC.^69,73^ The mzML data were deconvoluted to a MSALIGN file using TopFD (http://proteomics.informatics.iupui.edu/software/toppic/; version 1.6.5) with a maximum charge of 30, a maximum mass of 70,000 Da, an MS1 S/N ratio of 3.0, an MS2 S/N ratio of 1.0, an *m*/*z* precursor window of 3.0, an *m*/*z* error of 0.02 and HCD as fragmentation.^74^ The final sequence annotation was performed with TopPIC (http://proteomics.informatics.iupui.edu/software/toppic/; version 1.6.5) with a decoy database, maximal variable PTM number 3, 10 ppm mass error tolerance, 0.01 FDR cutoff, 1.2 Da PrSM cluster error tolerance, and a maximum of 1 mass shifts (±500 Da), and a combined output file for the nonreduced and reduced samples of a venom pool.^73^ Spectra were matched against the UniProt “Serpentes” database (ID 8750, reviewed, canonical and isoform, 2749 entries, last accessed on 11th October 2023 viahttps://www.uniprot.org/), manually validated, and visualized using the MS and MS/MS spectra using Qual Browser (Thermo Xcalibur 2.2 SP1.48). The XTRACT algorithm of Thermo Xcalibur was used to deconvolute isotopically resolved spectra.

### 2.6 Intact Mass Profiling and Peptidomics

The TD RAW data were manually screened in the Qual Browser (Thermo Xcalibur 2.2 SP1.48) for an overview of abundant intact protein and peptide masses. They were correlated to the previous peak annotation and identification by snake venomics as well as used for the counting of disulfide bridges between the nonreduced and reduced TD RAW samples. Spectra of multiple charges were isotopically deconvoluted by using the XTRACT algorithm of Thermo Xcalibur. Masses in this study are given in the deconvoluted average *m*/*z* (with *z* = 1), if not stated otherwise. Monoisotopic masses are also given with *z* = 1. In case of abundant non-TD-annotated peptides, masses were manually checked using Thermo Xcalibur Qual Browser (version 2.2 SP1.4), the peptide sequences were manually *de novo* annotated by the MS/MS spectra and the *m*/*z* peaks cross-confirmed by in silico fragmentation using MS-Product of the ProteinProspector (http://prospector.ucsf.edu, version 6.4.9).^75^

### 2.7 Proteome Quantification

The used quantification protocol is adapted from the common three-step “snake venomics” approach as summarized in Calvete et al. 2023 and our previous work.^76,77^ In short, the venom was quantified by RP-HPLC peak integrals (214 nm), densitometric quantification processed by Fiji^78^ and top3 ion intensities. Detailed formulas and calculations are placed in the Supporting Information under Additional Materials and Methods (Detailed proteome quantification).

### 2.8 Online Proteome Search

To identify relevant publications for the comparison of venom compositions the review of Damm et al. (2021) was used as template and database for Old World vipers (Squamata: Serpentes: Viperidae: Viperinae) venoms.^13^ We used the identical selection criteria parameters with two modifications. First, the genera, species, and subspecies taxa search were limited to Palearctic vipers of the genus *Vipera*, *Montivipera*, *Macrovipera* and *Daboia*, and the investigated time window was continued from first January 2021 until 31st December 2023.

### 2.9 Data Accessibility

MS proteomics data have been deposited via the MassIVE partner repository (https://massive.ucsd.edu/) under the bottom-up and top-down project names “Snake venom proteomics of seven taxa of the genera *Vipera*, *Montivipera*, *Macrovipera,* and *Daboia* across Turkiye/Turkey” with the data set identifiers “MSV000094228” and “MSV000094229”, respectively, as well as in the Zenodo repository (https://zenodo.org) under the project name “DATASET—Mass Spectrometry—Snake venom proteomics of seven taxa of the genera *Vipera*, *Montivipera*, *Macrovipera* and *Daboia* across Türkiye” with the data set identifier “10683187”.^79^

## 3 Results

**Figure 2:**
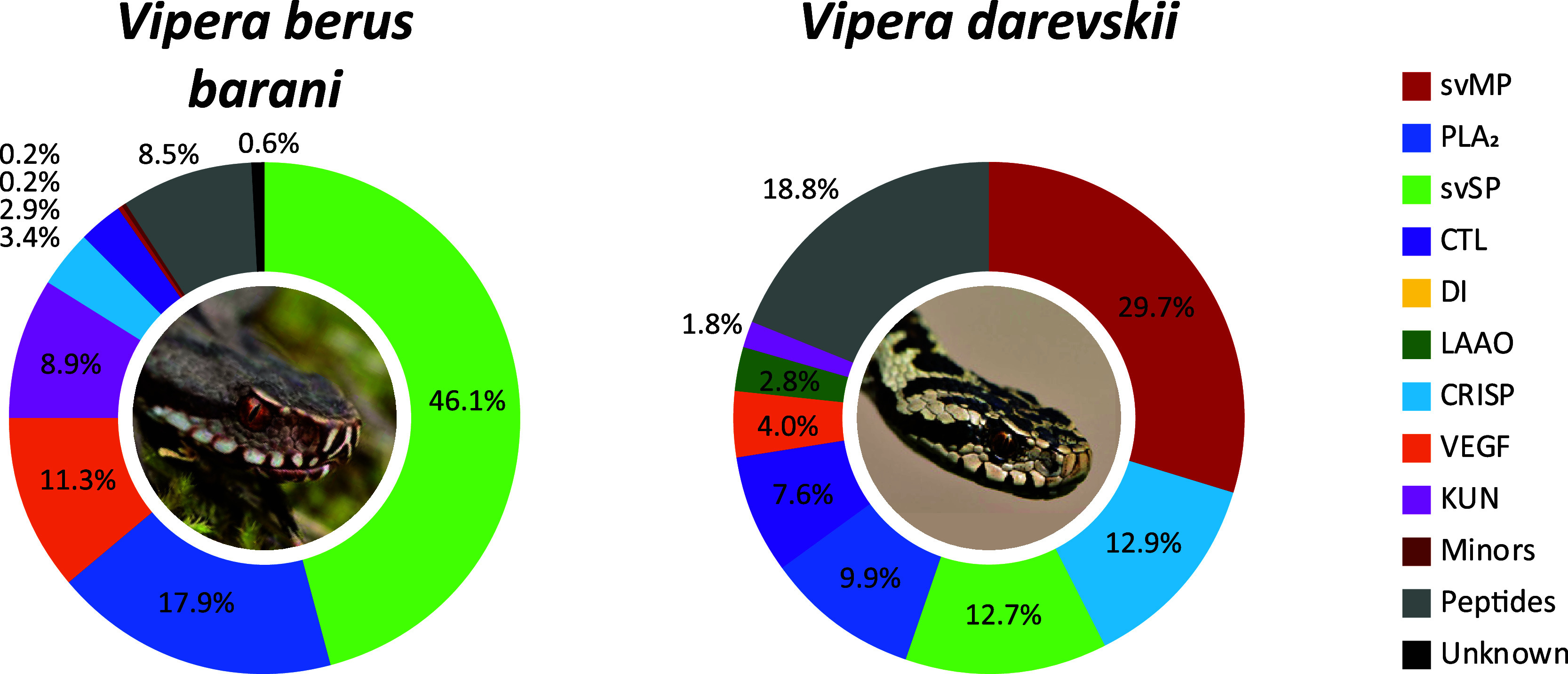
*Vipera* venom compositions of *V. b. barani* and *V. darevskii*. The venom proteomes of two *Vipera* taxa from Türkiye have been quantified by the combined snake venomics approach via HPLC (λ = 214 nm), SDS (densitometry) and MS ion intensity, including TD proteomics. Toxin families are arranged clockwise by abundances, followed by peptides (gray) and nonannotated parts of the venom (unknown, black). Images by Bayram Göçmen.

**Figure 3:**
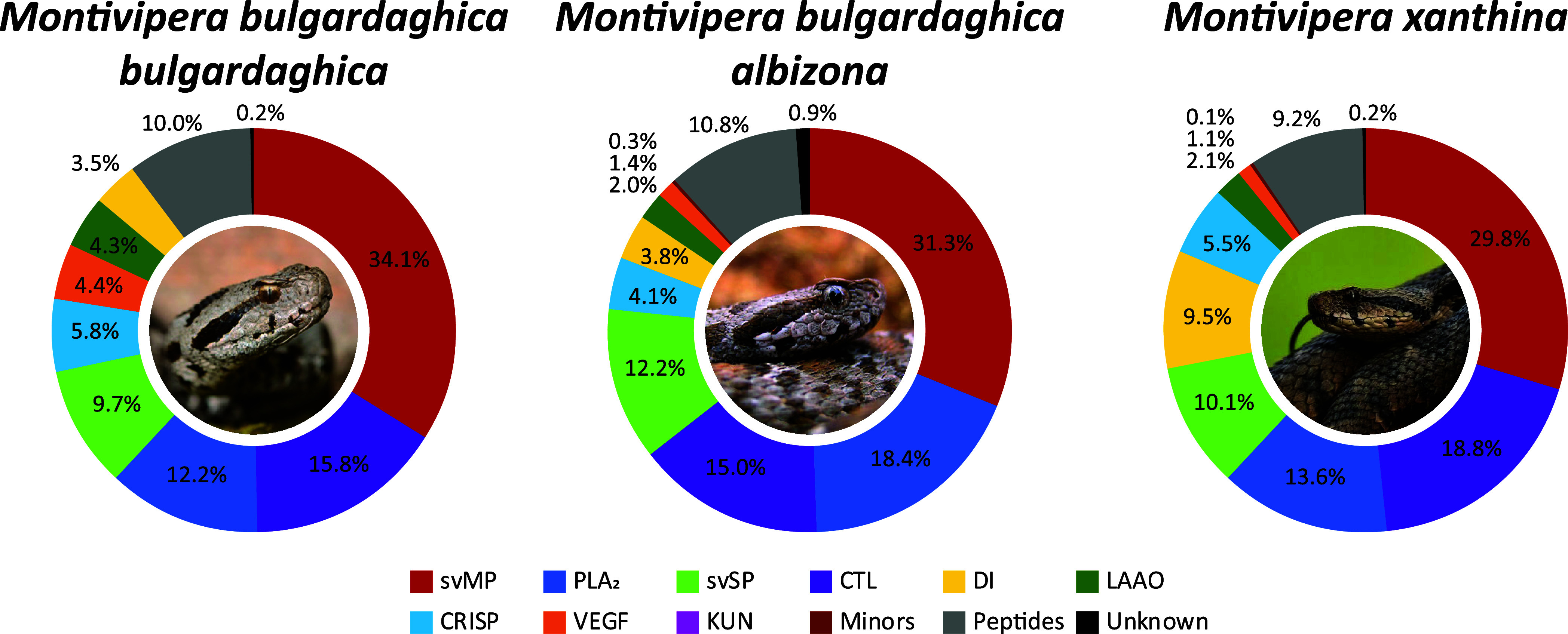
*Montivipera* venom compositions of *M. b. bulgardaghica*, *M. b. albizona,* and *M. xanthina*. The venom proteomes of three *Montivipera* taxa from Türkiye have been quantified by the combined snake venomics approach via HPLC (λ = 214 nm), SDS (densitometry) and MS ion intensity, including TD proteomics. Toxin families are arranged clockwise by abundances, followed by peptides (gray) and nonannotated parts of the venom (unknown, black). Images by Bayram Göçmen.

The venom proteomes of seven Palearctic viper taxa of Turkish origin were profiled by the snake venomics approach (Figures 2, 3 and 5, Supporting Information Figures S1–S7). For a comprehensive analysis each venom was additionally investigated by nonreduced and reduced top-down MS, including intact mass profiling and peptidomics. All identified toxins and homologues are in detail listed in the supplements (Supporting Information Tables S3–S9). Four venom proteomes represent first descriptions for these snake taxa (*V. b. barani*, *V. darevskii*, *M. b. albizona,* and *M. xanthina*), two have never been investigated before by extensive snake venomics for Turkish populations (*M. l. obtusa* and *D. palaestinae*) and one is an in-depth reanalysis in order to identify >20% of unknown proteins from a previous study (*M. b. bulgardaghica*, identical pool).^52^ In general, the seven proteomes largely conform to the previously proposed compositional family trends of toxins in viperine venoms.^13^ Accordingly, viperine venoms can be categorized into typical major-, secondary-, and minor toxin families. For those, the following abundance ranges were identified for the herein analyzed venoms:

- major toxin families: snake venom metalloproteinases (svMP, < 1–34%) including disintegrin-like/cysteine-rich (DC) proteins; snake venom phospholipases A_2_ (PLA_2_, 8–18%); snake venom serine proteases (svSP, 10–46%); C-type lectin-related proteins and snake venom C-type lectins (summarized as CTL, 3–20%),
- secondary toxin families: disintegrins (DI, 0–15%); l-amino acid oxidases (LAAO, 2–4%); cysteine-rich secretory proteins (CRISP, 0–13%), vascular endothelial growth factors F (VEGF, 0–12%), Kunitz-type inhibitors (KUN, 0–9%),
- minor toxin families: 5′-nucleotidases (5N, 0.1–0.8%); nerve growth factors (NGF, 0.3%); phosphodiesterases (PDE, 0.2%).

Members of rare families in Viperinae venoms, like glutaminyl cyclotransferases (EC 2.3.2.5) or aminopeptidases (EC 3.4.11.-), have not been detected in the herein studied venoms. In the following section, each snake venom composition will be described and the proteomes will be discussed on a genus-wide comparison. Furthermore, a variety of peptides (9–19%) have been observed in the venoms and will be highlighted later in detail separately.

### 3.1 *Vipera berus barani* and *V. darevskii*

With *V. b. barani* and *V. darevskii* two different taxa of the *Vipera* subclade *Pelias* have been analyzed in this study (Figure 2, Supporting Information Tables S3, S4, S10, S11, S17, S18). The *V. b. barani* crude venom HPLC profile lacks abundant peaks at *R*_t_ > 90 min and svMP are surprisingly underrepresented and correspond to only 0.2% of the venom (Supporting Information Figure S1). They were identified as members of the P–III subfamily and accordingly no DI were observed.

On the other side, the venom profile has a complex peak structure in the chromatogram between 75 and 90 min (F27–38) and svSP were identified as the most abundant toxin family. The fractions (F) F27–45 contain svSP of up to 32 kDa and the IMP revealed *m*/*z* 30,327.40 and *m*/*z* 30,909.67 as the most abundant average svSP masses. Both masses appeared in groups of peaks, based on the variable *N*-glycosylation with mass shifts of Δ203 Da and Δ406 Da, indicating at least two *N*-acetylhexosamines (HexNAc, 203.08 Da). By BU, nikobin was identified as homologue in most of the fractions. The remaining svSP were identified as homologues to the hemotoxic factor V-activating enzyme (RVV-V, *Daboia siamensis*) or svSP homologue 2 (*M. lebetinus*).

A combination of basic, neutral and acidic PLA_2_ (18%) formed the second most abundant toxin family and all PLA_2_ in the *V. b. barani* venom were identified as neurotoxic homologues via BU proteomics.^80,81^ By TD proteomics proteoforms of ammodytin (*m*/*z* 13,553.88, 13,676.39, 13,692.84) and ammodytoxin (*m*/*z* 13,742.19, 13,773.18, 13,856.25) were annotated and the PLA_2_ conserved seven intramolecular disulfide bridges could be confirmed (Supporting Information Table S17). The following most abundant toxin families were VEGF (11%), mostly vammin-1′ related, and KUN (9%) formed by a single serine protease inhibitor ki-VN (*m*/*z* 7594.47) with three TD confirmed disulfide bridges. Further toxin families are CRISP (3%), with a single dominant band in F24/25, CTL (3%), PDE (0.2%) and LAAO in small traces (band 44c). Abundant peptides signals have been identified by MS2 as pERRPPEIPP (*m*/*z* 1072.59) and pERWPGPKVPP (*m*/*z* 1144.62), beside two tripeptidic svMP inhibitors (svMP-i) pEKW (*m*/*z* 444.22) and pERW (*m*/*z* 472.23).

The second *Vipera* venom investigated in this study stems from *V. darevskii*. It largely follows the classical Viperinae composition and is characterized by high abundances of svMP (30%, P–III svMP only), PLA_2_ (10%), svSP (13%), and CTL (8%) as major toxin families.

The main PLA_2_ are acidic homologues, such as myotoxic ammodytin L1, as well as MVL-PLA2 and VpaPLA2 from *Daboia* and *Macrovipera* species. One-third of the svSP shared the highest similarities with anticoagulant active homologues of *Vipera ammodytes*, while the remaining 9% (*R*_t_ > 80 min), were matched to sequences from *V. berus* (nikobin). The CRISP (13%) toxins are second most abundant, and interestingly, a strong signal for a CRISP fragment has been observed with a monoisotopic mass of *m*/*z* 6414.61, eluting at 11 min in the nonreduced, nondigested venom. Its reduced monoisotopic signal of *m*/*z* 6424.68 could be annotated by TD as the *C*-terminal fragment of CRVP_VIPBN, a CRISP from *V. berus nikolskii*, with a single oxidation (+15.99 Da). The mass shift of Δ10.065 Da indicates five disulfide bridges through all ten Cys in the sequence. Several further secondary toxin families were identified, like VEGF (4%), LAAO (3%) and KUN (2%), but no DI nor any minor or rare were detected. The peptides (19%) are dominated by a single svMP-i (pEKW) fraction with over 11% of the whole venom proteome of *V. darevskii*. Furthermore, 3% could be assigned to the de novo annotated peptide pENWPGPK (*m*/*z* 809.39).

### 3.2 *Montivipera bulgardaghica* ssp. and *M. xanthina*

**Figure 4:**
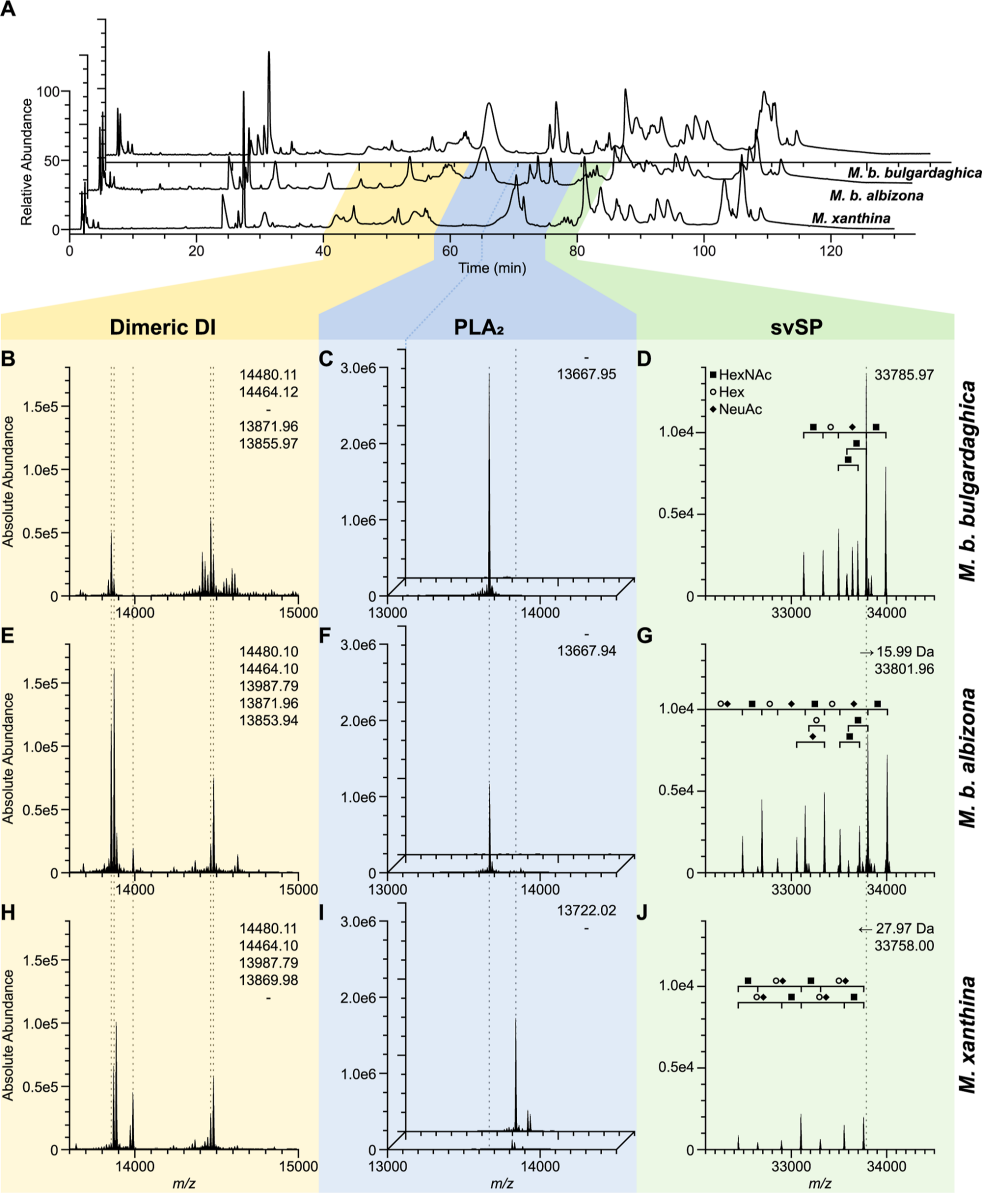
Venom profiles of three mountain vipers (*Montivipera*) and comparison of abundant toxins. (A) Chromatogram of the venoms from *M. b. bulgardaghica* (top/back; B–D), *M. b. albizona* (middle; E–G), and *M. xanthina* (bottom/front; H–J) with λ = 214 nm. (B–J) Exemplary main toxin families were investigated by nonreduced intact mass profiling (IMP) at their corresponding top-down proteomics retention times set in correlation to the snake venomics HPLC profile. The deconvoluted main toxin masses (dashed lines) are compared for five dimeric DI (B,E,H at 11.4–15.2 min IMP RT) and two PLA_2_ at two different times (C,F,I at front 15.3–18.0 min and back 18.0–19.7 min IMP RT). Begin of the second PLA_2_ time windows in (A) is connected (dark blue line) the corresponding IMP (back of C,F,I). A svSP (D,G,J at 20.5–21.2 min IMP RT) shows small mass shifts but similar glycosylation components: HexNAc (*N*-acetylhexosamines, filled square), Hex (hexose, circle), NeuAc (*N*-acetyl neuraminic acid, filled rhombus). Abbreviations: DI, disintegrins (yellow); PLA_2_, phospholipase A_2_ (blue); svSP, snake venom serine protease (green).

The genus of *Montivipera* is represented by two *M. bulgardaghica* subspecies (*M. b. bulgardaghica*, *M. b. albizona*) and *M. xanthina* (Figure 3, Supporting Information Tables S5–S7, S12–S14, S19–S21). The profiles between the *M. bulgardaghica* ssp. had higher similarities in the chromatograms of the first 75 min compared to *M. xanthina*, while eluting profiles between 80 to 110 min of all three venoms had exhibited striking similarities (Figure 4).

In all three *Montivipera* venoms different svMP (30–34%) dominate, mostly P–III svMP to a smaller extend of DC proteins (2–4%), followed by CTL (15–19%) (Figure 3). Each venom had three main fractions between 82 and 104 min with abundant CTL bands in the reduced SDS PAGE consistent to their multimeric structure.^82^ The observed tryptic peptides sequences were homologue to *M. lebetinus* toxins in all three snakes: Snaclec A11/A1/B9 (82 min), Snaclec A16/B7/B8 (88 min), and C-type lectin-like protein 3A (104 min).

The PLA_2_ (12–18%) differ between the species. The acidic phospholipase A_2_ Drk-a1 homologue, from *Daboia russelii*, is the main representative in both, *M. b. bulgardaghica* (11%) and *M. b. albizona* (12%) (Figure 4C,F,I). The PLA_2_ were detected in a single dominant peak at *R*_t_ 62 min, at which the *M. xanthina* chromatogram had only a flat broad signal (F22). In the *M. xanthina* composition this fraction has been identified by BU as a coelution of NGF (0.1%) and PLA_2_ (1.3%). Its main PLA_2_ eluted a few minutes later at ∼70 min forming a strong signal (F23–25), which in turn was absent in the first two profiles. In *M. xanthina* a different main acidic PLA_2_ homologue with *m*/*z* 13,722.02 has been observed. It represents over 8% of the whole venom (Figure 4C,F,I). Basic PLA_2_ were only be detected in traces within the two *M. bulgardaghica* subspecies.

Within all three HPLC profiles a group of close eluting peaks has been detected at <80 min, which is typical for svSP in viper venoms bearing an extensive glycosylation. The main svSP masses differ within the genus of *Montivipera*, but are closely related with mass shifts of Δ15.99 Da (O) between *M. b. bulgardaghica* and *M. b. albizona*, and Δ27.97 Da (CO) between *M. b. bulgardaghica* and *M. xanthina* (Figure 4D,G,J). All three had peak patterns of same distances and revealed so similar consecutive glycosylations, with observed mass shifts of Δ203 Da (HexNAc, 203.08 Da), Δ162 Da (hexose Hex, 162.06 Da), and Δ291 Da (*N*-acetyl neuraminic acid NeuAc, 291.10 Da) (Figure 4D,G,J).

Secondary toxin families were identified at lower abundances: DI (4–10%), CRISP (4–6%), LAAO (2–4%), and VEGF (1–4%) of which all belong to the vammin/ICCP-type,^83^ but no KUN have been detected in any *Montivipera* venom. In total, 11 different abundant masses could be identified as heterodimeric DI around 14 kDa, and while monomeric DI of various lengths from 4 to 8 kDa are known to appear in viper venoms, none of these have been observed in the herein analyzed *Montivipera* venoms. *M. xanthina* showed with 9.5% more than twice the amount of DI than *M. b. bulgardaghica* (3.5%) and *M. b. albizona* (3.8%). Only two abundant dimeric DI are shared across all three venoms (Figure 4B,E,H), and TD revealed the two subunits as homologues of *M. lebetinus* and *Eristicophis macmahoni* The other ten dimeric DI were either detected in two of the three vipers, or unique for one of them. For example, both *M. bulgardaghica* ssp. shared *m*/*z* 13,871.96, while *m*/*z* 13,987.79 has been only observed for *M. b. albizona* and *M. xanthina* (Figure 4B,E,H).

The three CRISP containing peaks eluted contemporaneous in the *Montivipera* venoms at *R*_t_ = 70 min, with different main representative masses. For minor toxins only 5N (0.3%) were annotated by BU in the venom of *M. b. albizona* and NGF (0.1%) in *M. xanthina*.

The three *Montivipera* venoms contain a similar peptide part of around 10% and the svMP-i pEKW, pERW, and pENW (*m*/*z* 430.17) could be identified in all of them as abundant components. The decapeptide pENWPSPKVPP (*m*/*z* 1132.55) and two C-terminal truncated peptides were also prominent in each *Montivipera* peptidome as well as the glycine-rich peptide pEHPGGGGGGW (*m*/*z* 892.37).

### 3.3 *Macrovipera lebetinus obtusa*

**Figure 5:**
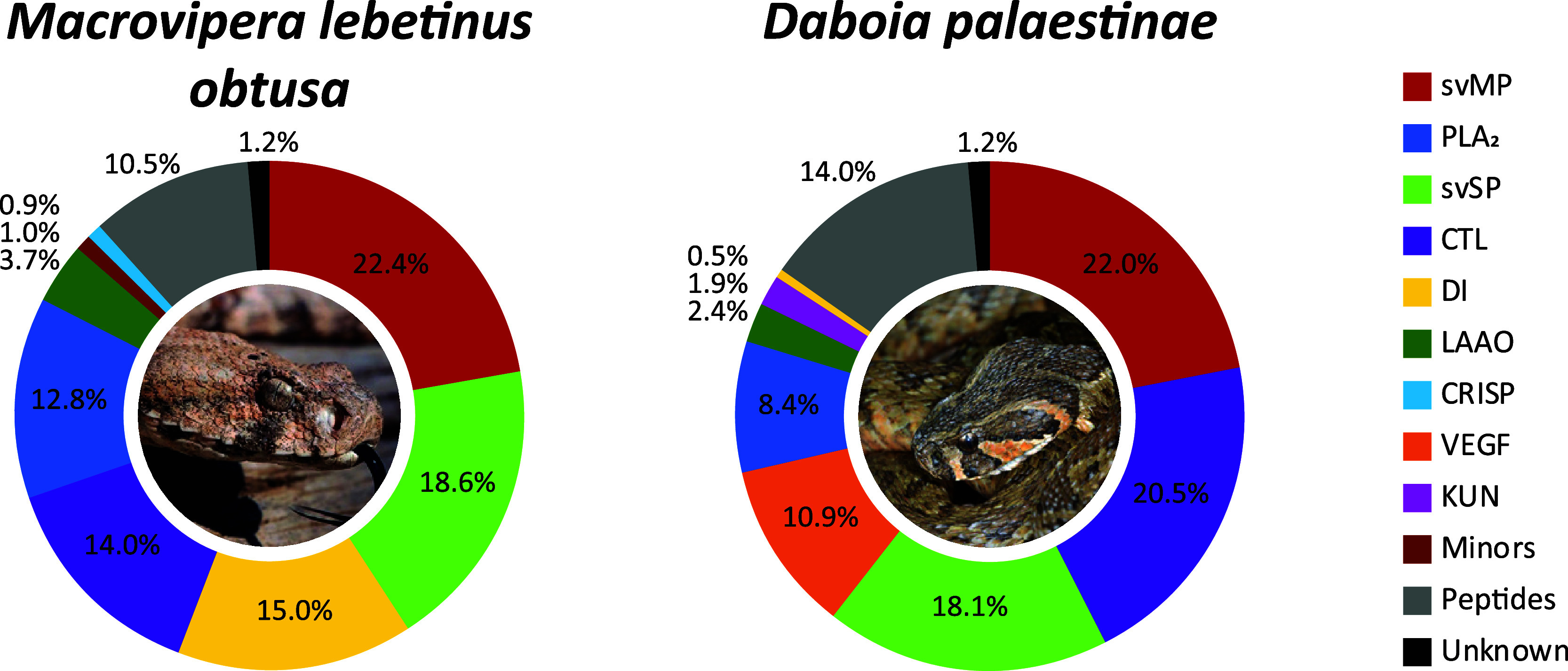
*Macrovipera*and *Daboia*venom compositions of *M. l. obtusa* and *D. palaestinae*. The venom proteomes of one *Macrovipera lebetinus* subspecies and one *Daboia* species from Türkiye have been quantified by the combined snake venomics approach via HPLC (λ = 214 nm), SDS (densitometry), and MS ion intensity, including TD proteomics. Toxin families are arranged clockwise by abundances, followed by peptides (gray) and nonannotated parts of the venom (unknown, black). Images by Bayram Göçmen (*Macrovipera*) and Mert Karış (*Daboia*).

The third Palearctic viper genus analyzed was *Macrovipera* represented by the venom of *M. l. obtusa* (Figure 5, Supporting Information Tables S8, S15, S22). Its major toxins, including DI, forming 83% of the venom and are mostly composed of svMP (22%), with P–I (2%) and P–III svMP (12%). The DC proteins, or P–IIIe svMP subfamily, account for >8% of the venom. The most abundant P–III svMP was the heavy chain of the coagulation factor X-activating enzyme VLFXA. It forms a heterotrimeric complex with the CTL light chains 1 and 2, annotated in F38 and F40. Further abundant svMP include the apoptosis inducing VLAIP-A/B (P–III) and lebetase (P–I). The svSP (19%) consist of different toxins, that has been previously described from the *Macrovipera* genus and a majority of the tryptic peptide sequences originated from the coagulant-active lebetina viper venom FV activator (VLFVA or LVV-V), followed by the α-fibrinogenase (VLAF), VLP2, and VLSP3. The third most common toxin family are DI (15%) and we could identify more than ten dimeric DI masses (Supporting Information Table S24). The main DI subunits are from known *Macrovipera* toxins, such as lebein-1, VB7A, VLO4, VLO5, VM2L2, or lebetase. This high variety of dimeric DI is a result of mass shifts (oxidation Δ15.99 Da, hydration Δ18.01 Da) and terminal amino acid truncations. No monomeric DI were observed.

The remaining major families are CTL (14%), with the two previously mentioned VLFXA light chains as well as only two PLA_2_ (13%), eluting around 80 min in the HPLC profile. They were identified as acidic phospholipase A_2_ 1 (6.4%; *m*/*z* 13,662.79, nonred.) and A_2_ 2 (6.4%, *m*/*z* 13,644.79, nonred.). Additionally, LAAO (4%), CRISP (0.9%), NGF (0.8%), and PDE (0.2%) were detected as less dominant toxin families.

The venom profile of the analyzed *M. l. obtusa* is dominated by one peptide containing peak (F5), with 9% of the whole venom formed by pEKW and its 2M + H^1+^ ion of *m*/*z* 887.44. Further abundant peptides are pEKWPSPKVPP (*m*/*z* 1146.63) and pEKWPVPGPEIPP (*m*/*z* 1327.71).

### 3.4 *Daboia palaestinae*

The last Viperinae genus *Daboia* is represented by *D. palaestinae*. Its venom is largely composed of svMP (22%) with only P–III svMP (16%) and DC proteins (6%), as well as an abundant amount of CTL (21%) (Figure 5, Supporting Information Table S9, S16, S23). The earlier eluting CTL at *R*_t_ = 82 to 88 min (F28–33) have been annotated as homologues to *M. lebetinus*, while the later (*R*_t_ > 90 min) are related *D. palaestinae* toxins. The third abundant toxin family, svSP (18%), is described by different fibrinogenases and plasminogen activators. The HPLC venom profile lacks any dominant peak between *R*_t_ = 60 and 75 min and no CRISP were observed and PLA_2_ (8%) were only described within F26/27.

Secondary toxin families in the venom of *D. palaestinae* are VEGF (11%), mainly homologue to VR-1 from *D. siamensis*, LAAO (2%) and KUN (2%). The *m*/*z* 7722.582 signal was identical to then KUN serine protease inhibitor PIVL from *M. l. transmediterranea*. The only DI (0.5%) is the small KTS sequence containing viperistatin with *m*/*z* 4469.84 and four TD confirmed disulfide bridges. No minor or rare toxin families were observed within the Turkish *D. palaestinae* venom.

The peptidic part (14%) includes as main representatives, two svMP-i (pEKW, pENW) already detected within the other viper venoms of this study. But while no pERW mass has been observed, several related sequences could be annotated, such as pERWPGPKVPP (*m*/*z* 1144.63) and pERWPGPELPP (*m*/*z* 1159.59).

## 4 Discussion

**Figure 6:**
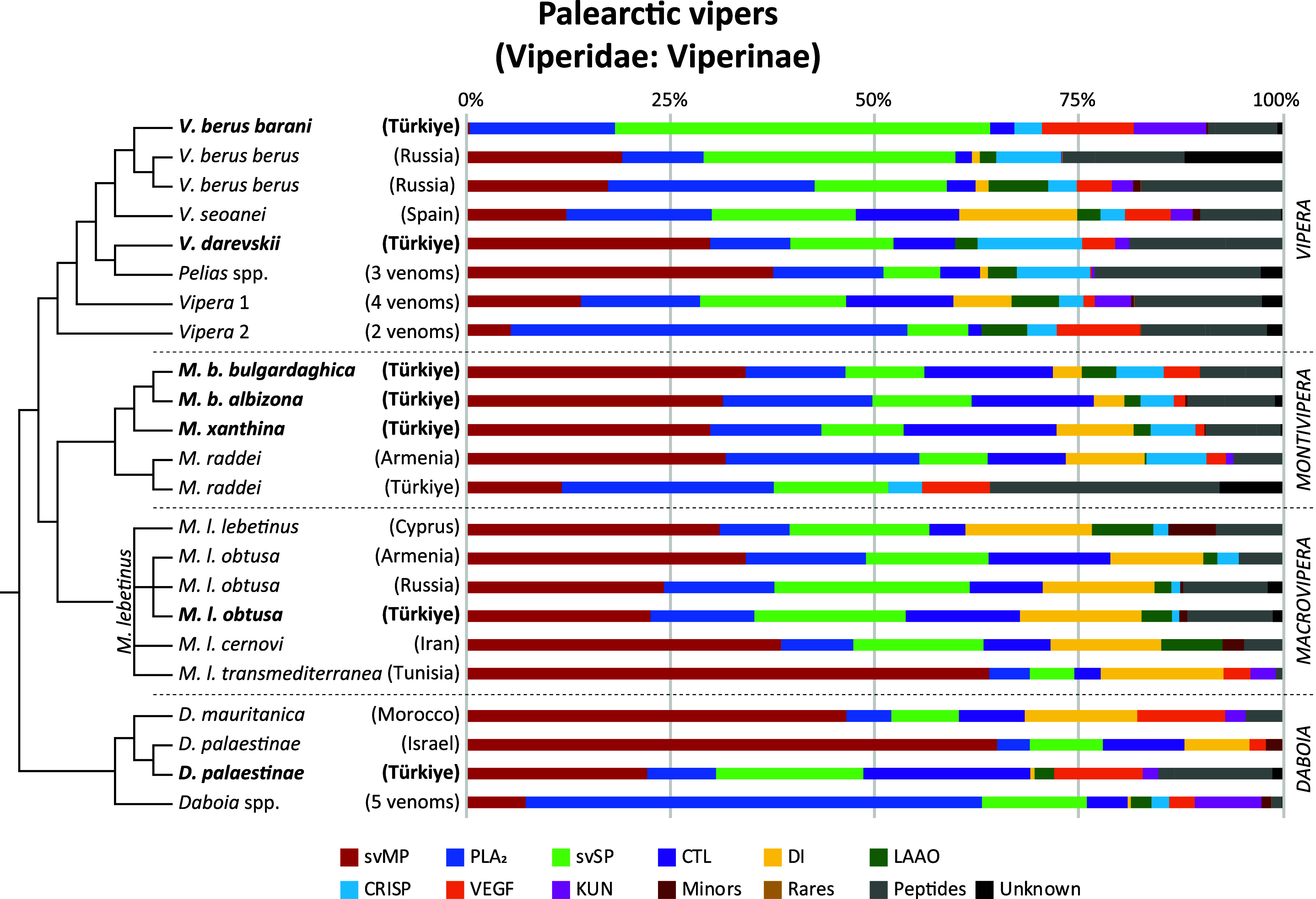
Snake venomics of Palearctic viper venom proteomes. Overview of all four genera (*Vipera*, *Montivipera*, *Macrovipera,* and *Daboia*) and updated to Damm et al. (2021). The 33 comparative proteomics data of 15 different Viperinae species including subspecies are lined up phylogeny-based. Origins of investigated specimen are reported in brackets. Numbers represent investigations of >1 venom proteomes. Venoms from this study are in bold. Schematic cladograms of the phylogenetic relationships based on Freitas et al. (2020).

To gain better insights into the venom variations and the potential impact of medical significance of Palearctic vipers, we aligned the data of the seven vipers in a genus-wide comparison (Figure 6). For this purpose, we updated the previous venomics database of the full Old World viper subfamily (Viperinae) from Damm et al. (2021) and added additional snake venomics studies of Palearctic vipers until the end of 2023, searched by identical parameters.^13^

### 4.1 *Vipera*—Eurasian Vipers

With more than 20 species the Eurasian vipers (genus *Vipera*) are the most diverse group of all Old World vipers and can be split into three major clades: *Pelias*, *Vipera* 1, and *Vipera* 2.^15^ While in Europe snakebite envenoming is an neglected health burden, even so over 5500 case have been reported in total, several species are of medical relevance, i.a. *V. berus*, *V. ammodytes,* and *Vipera aspis*.^12,84,85^

Above all, the adder *V. berus* is of particular interest for venom research, as it is still completely unknown to what extent a venom composition changes within such extremely large distribution range. Various factors such as genetic isolation and different habitats over several thousand kilometers across different climate zones with variable prey can have an unforeseen influence on the venom composition and make it impossible to predict variations.^28^ Therefore, it is surprising that relatively little is known about venom variations, both of nominal *V. berusberus* and the multitude of subspecies (*barani*, *bosniensis*, *nikolskii*, *marasso*_*,*_ and *sachalinensis*).^17,86^ Only four venomic data sets have been reported beside our *V. b. barani* venom, with two Russian *V. b. berus* analyzed by snake venomics in addition to the related *Vipera seoanei*.^13,77,87−90^

Other studies over the past decades were based on single toxin isolation and characterization, or physiological effects.^86^ The two Russian *V. b. berus* snake venomics studies show the remarkable differences to the herein presented *V. b. barani* venom as svMP are nearly missing and is dominated by svSP, VEGF and KUN forming over 66% of the proteome (Figure 6). The only other *Vipera* described to harbor comparatively low svMP levels are *V. ammodytes montandoni* (1.8%) and the close related *V. b. nikolskii* (0.7%).^13,56,90^ While high svSP contents are known for other Viperinae, like *Bitis* (15–26%), *Cerastes* (7–25%), or *Macrovipera* (5–24%) so far, only the venom of Russian *V. b. berus* with 30% svSP has been described with an increased svSP content.^13^ With 46% svSP the composition of the Turkish *V. b. barani* renders unique among so far quantified Old World viper venoms. Its most prominent protein, Nikobin, is, like most svSP, a glycoprotein with unknown glycosylation pattern and putative hemotoxic activity.^91,92^ Sequences of the proteins show three *N*-glycosylation recognition sites, which high potential variability would explain the complex peak pattern observed for the *V. b. barani* venom profile. It is questionable to what extent the clinical manifestations would be similar, as there is only one suspected case report of this subspecies to date.^93^ In addition to local swelling, and hyperemia, there were clear neurological symptoms with pronounced diplopia and ptosis. The bites of *V. berus* have a broad spectrum of potential effect, and is often per se defined as cyto- and hemotoxic with pro- or anticoagulant inducing effects and blood factor X activators.^86,94^ However, one problem is that the neurotoxic effects of *V. berus* envenoming are poorly documented in comparison to the amount of bite cases, but known for the other two medical relevant species, *V. aspis* and *V. ammodytes*.^23,95−99^ PLA_2_, such as presynaptic ammodytoxin isoforms and postsynaptic isoforms of aspin and vipoxin, are most likely responsible for these effects.^88,100,101^ This toxin family could be detected in all *V. berus* venom proteomes in varying abundances as well our *V. b. barani*.^13,90^ The impact of the extremely high svSP content in *V. b. barani* might be accompanied by strong effects on coagulation pathways and platelet aggregation like in other vipers.^92,102^ This shows that the venoms of the Eurasian adders are far more complex than previously investigated and thus represents an important subject for future venom research with a high relevance for the therapeutic treatment and specimen/population selection for antivenom development. It needs to be noted, that none of the antivenoms has been assessed by the WHO until now, but are registered by competent national authorities and many vipers of lower medical interest are often not tested, therefore the antivenom efficiency against many of those taxa remains unknown.^18,103,104^

The taxonomically complex *Vipera* genus has several taxa with nearly no knowledge about bite consequences and their venom composition and pathophysiology.^15,105^ Identified toxins within those neglected vipers often show homologies to highly active compounds of medically relevant taxa, such as *V. ammodytes* and *M. lebetinus*. One example is the here described *V. darevskii* venom, that is mainly dominated by svMP and confers to the classical Viperinae arrangement of major and secondary toxin families. Whether the described truncated *C*-terminal CRISP is an artificial cleavage product of the main toxins or an independently functional toxin cannot be determined from its sequence alone. Nevertheless, it is striking that it represents a self-contained and structurally stabile subdomain with five disulfide bridges, referred to as the Cysteine-Rich Domain (CRD) or Ion Channel Regulatory (ICR) domain.^106^ This domain contains the ShKT superfamily like sequence known from highly potent small venom peptides produced by anemones with a strong effect on potassium channels.^107^ Similarly, in snake venoms other C-terminal subdomains are known to have evolved into independent toxins, such as DI and DC proteins from svMP.^108−110^

Additionally, such neglected taxa have similar large proportion of peptides, consisting of bradykinin-potentiating peptides (BPP) and natriuretic-related peptides, which even at low concentrations can have serious effects on the corresponding physiological systems. With high homology or even identical sequences to the BPP of pit vipers, as the most famous *Bothrops jararaca*, suggests that these peptides may also be responsible for corresponding responses in Palearctic vipers as herein described for all four genera, and discussed later in detail.^111^

### 4.2 *Montivipera*—Mountain Vipers

The mountain vipers (genus *Montivipera*) are divided into two clades, the Ottoman vipers *M. xanthina* including *M. bulgardaghica* and the *M. raddei* complex. In comparison to the other three Palearctic viper genera, little is known about their venoms and the clinical consequences of a bite,.^52,112,113^ Reported bites are from Türkiye, Armenia, Lebanon and Iran and describe symptoms reaching from local effects such as extensive blistering, local edema and necrosis up to coagulopathy and leucocytosis, and in two cases with lethal consequences.^112,114^

Our mass spectrometric analysis revealed that the venoms of the three examined *Montivipera* spp. are relatively similar. A genus-wide comparison showed, that also the venom profile of the Armenian *M. raddei* has also a similar composition, with the Turkish *M. raddei* venom surprisingly divergent (Figure 6). These include nearly 30% peptide content and 8% of unknown identity.^52,115^ Our discovery of PLA_2_, VEGF and CTL homologues to toxins of *D. russelii*, *D. siamensis*, *M. lebetinus,* and *V. ammodytes* in all three *Montivipera* venoms emphasizes their potential hazardous nature. The intravenous murine LD_50_ for Iranian *Montivipera latifii* and *M. xanthina* was determined to be < 0.5 mg/kg, in the same range as the Caspian cobra *Naja oxiana*, saw-scaled viper *Echis carinatus* and *M. lebetinus* (determined in μg venom per 16–18 g mouse), analogous to the results of a comparison of 18 different Palearctic viper taxa.^116,117^ The similarities found for such snakes of medical relevance indicates that the genus *Montivipera* is of comparable danger. Consequently, bites must be treated with equal caution particularly at the hemo- and neurotoxic level. This is exemplified by several *Montivipera* spp. venoms with potent anticoagulant effects on human plasma.^118^ The WHO lists only a few antivenoms with *Montivipera* taxa as immunizing venom species, namely *M. xanthina* and *M. raddei*, including the previously mentioned Inoserp Europe.^12,18,117^ Therefore, it remains questionable whether such antivenoms are effective against the lesser known *Montivipera* species, especially since some venom are similar at the intragenus level (here four of five proteomes), but can be strongly variable at the species level, like in *M. raddei* (Figure 6).

### 4.3 *Macrovipera*—Blunt-Nosed Vipers

The blunt-nosed vipers *Macrovipera* are widely distributed in the Middle East.^119,120^ Its most widespread representative, *M. lebetinus*, including several subspecies, can be found in over 20 countries and is by the WHO listed as highly medical relevant in more than half it.^18,20,21^ A detailed genus-wide comparison of all blunt-nose vipers venoms has been published recently in tandem with a detailed biochemical and pharmacological overview of *M. lebetinus* ssp. toxins.^121,122^ Thus, these aspects will only be briefly discussed here.

The overall composition of our Turkish *M. l. obtusa* venom mirrors that of the Armenian and Russian *M. l. obtusa*, and also the other subspecies (*M. l. lebetinus* and *cernovi*) share similar compositions, with the *M. l. cernovi* venom showing the largest divergence (Figure 6). The taxonomically debated African subspecies *M. l. transmediterranea* is a clear outlier, with a noteworthy increased proportion of svMP. With its VEGF and KUN, the venom is more similar to *Daboia mauritanica*, which also occurs in the areas of North Africa. It should be emphasized that *Macrovipera* has the largest DI amount of the four genera with a consistently high content of 11–16%, independently to the DI subfamily composition. Although the expected monomeric, KTS sequence containing short DI obtustatin was originally characterized as high abundant toxin of *M. l. obtusa* (unreported local origin) with 7% of the whole venom proteome, no short nor monomeric DI has been described until now for any Turkish and Iranian *Macrovipera* venom,^121,123^ while several R/KTS DI are known from other Viperidae venoms, including recently *Vipera*.^124,125^ Similarly, the venoms of another Turkish *M. l. obtusa* location and an Iranian *M. l. cernovi* lack small DI, while the Russian and Armenian *M. l. obtusa* contain them.^121^ This indicates that the subfamily of monomeric R/KTS DI is diversely distributed even within the genus *Macrovipera*. A detailed understanding of DI heterogeneity is of clinical importance and accordingly, this aspect demands further investigation in the future. A sequence clustering showed, that dimeric and short DI are the closest related snake venom DI subfamilies and might be a hint for this shift in their composition.^126^ A previous study, focusing on the Milos viper (*Macrovipera schweizeri*, recognized as a subspecies of *M. lebetinus* by several authors) and three *M. lebetinus* ssp. showed similar HPLC, SDS and bioactivity profiles.^121^ On the clinical side, it is therefore to be expected that the symptoms across the investigated *M. lebetinus* ssp. localities might be similar to effects on hypotension, hemorrhage and strong cytotoxicity leading to necrosis.^127,128^ On the other side, the geographic distribution of *Macrovipera* is large and includes an array of environments, so it is difficult or even impossible to predict venom variation, equal to the earlier mentioned *V. berus*.

### 4.4 *Daboia*

The *Daboia* spp. ranks among the most medically significant snake lineages. They consist of a venom-wise understudied western Afro-Arabian group (*D. mauritania*, *D. palaestinae*), and the eastern Asian group, with *D. russelii* belonging to Indians “Big Four”. About 18 venom proteomes have been published for *D. russelii*, in addition to the 11 of the closely related *D. siamensis*, formerly *D. russelii siamensis* (Supporting Information Table S2). *Daboia* is a prime example for the effect of biogeographic venom variation, with notable effects on the limited antivenom usability across an entire distribution area.^129^ This underline how not only on a genus-wide but also on intraspecific venom variations manifest into a problem of high therapeutically and scientific interest.

The venom of *D. palaestinae* has been investigated three times in a venomics context, of which one has been quantified by peak intensities of a shotgun approach and two by snake venomics, but at different wavelength (230 nm versus 214 nm this study).^130,131^ The other two were of Israeli origin, while this study based on the recently described Turkish population. Even if not all three studies can be directly compared, the two snake venomics approaches (Israel, Türkiye in this study) show already considerable differences (Figure 6). While the Israeli sample, similar to the *D. mauritanica*, is dominated by svMP (65%) and contains a relevant amount of DI (8%), the Turkish venom shows a rather unusual composition, as previously described in detail. In particular, the lack of DI and the high level of VEGF distinguish it from the Israeli proteome from 2011.^130^ The Israeli shotgun composition from 2022, on the other hand, even lists svSP as the main toxin group, followed by CTL and PLA_2_, while the svMP only make up a marginal proportion of the identified peptides (3%).^131^ With these different analytical methods in mind, it shows clearly that all three *D. palaestinae* venoms have a significantly different composition. While Senji Laxme et al. (2022) reported in a direct comparison that the Israeli *D. palaestinae* is svSP and the Indian *D. russelii* svMP dominated, Damm et al. (2021) showed in a proteomic meta-analysis that *Daboia* venoms are more split into an Afro-Arabian and an Asian *Daboia* venom clade.^13,131^ They are dominant in SVMPs with DI in the western clade, while PLA_2_ rich in the eastern clade, in contrast to the *D. palaestinae–russelii* comparison carried out by Senji Laxme et al. (2022). However, the herein newly reported venom composition of the Turkish population does not exactly fit to either assignment. To what extent the venoms of *Daboia*, and *D. palaestinae* in particular, are really that multivariant or artifacts of different analysis methods needs to be clarified in future.

Especially the strongly reduced svMP and DI in the Turkish venom, as well as the increased proportion of svSP and VEGF might have severe influence on the degree of clinical symptoms, since a previous bioactivity-guided study on the hemotoxic properties revealed that *D. palaestinae* venom from different localities (twice Israel, once unknown) had evident variation in its activity across most of the tested assays.^132^

### 4.5 Small Venom Peptides of Palearctic Vipers

The proteomic landscapes of snake venoms are intensively investigated and reviewed.^13,76,133^ However, the knowledge about their lower molecular weight, peptidic compounds are more restricted. While several of the larger peptide families, with sizes up to 9 kDa, are often reported as toxin families on their own (such as three-finger toxins (3FTx), KUN, DI, or crotamine), components below 4 kDa are largely neglected.^134,135^ A variety of BPP, which were with their strong hypotension activity a template for Captopril, are known from Crotalinae venoms, but only few studies looked into the peptidome of Viperinae.^13,111^

Our rigorous MS profiling allowed us for the first time, to identify an array of low molecular weight peptidic components from the seven herein analyzed taxa. As mentioned in the previous part, i.e. KUN and different DI are well-known for viperine venom and were usually identified in our analyzed samples. While in *Vipera*, the peptide fraction fluctuated profoundly between taxa (ranging from 9 to 19%), the peptide landscape was more consistent in all three *Montivipera* spp. at 9–11%. *M. l. obtusa* and *D. palaestinae* showed 10–13%, respectively (Figure 6).^13^ Nevertheless, their compositions and the relative abundances of certain peptides differed strongly between the venoms and also within the same genera. Those identified peptides potentially originate from BPP and natriuretic peptide (NP) precursors, that can include repetitive svMP-i tripeptides and poly-His-poly-Gly (pHpG) sequences.^136^ A key element of most such peptides is the *N*-terminal pyroglutamate (pE), formed by glutaminyl cyclotransferases, which have been identified several times in viper venoms.^13^ The overall comparison showed strong similarities in the appearance of abundant peptides within *Montivipera*, the peptidome of which seems related to that of the *M. l. obtusa* (Table 1). Surprisingly, the peptidome of *V. b. barani* is more similar to *D. palaestinae*, than the taxonomically closer *V. darevskii*.

**Table 1:** Peptidomics of svMP-i, BPP and NP of Palearctic Vipers^a^

sequence	MH^1+^(mono) *m*/*z*	mass with *z* = 2 (mono) *m*/*z*	*V. b. barani*	*V. darevskii*	*M. b. bulgardaghica*	*M. b. albizona*	*M. xanthina*	*M. l. obtusa*	*D. palestinae*	notes
**Lys (K) Related**										
pEKW	444.224		•	•	•	•	•	•	•	2MH^+1^ (*m*/*z* 887.441)
pEKW_ox_	460.219		•	•	•	•	•	•	•	Trp oxidation
pEKWP	541.277				•	•	•	•	•	
pEKWPSPK	853.457	427.232			•	•		•	•	
pEKWPSPKVPP	1146.631	573.819			•	•		•		
pEKWPVPGP	891.472	446.240			•	•	•	•		
pEKWPVPGPEIPP	1327.705	664.356			•	•	•	•		
pEKWPM_ox_PGPEIPP	1375.672	688.340							•	Met oxidation
pEKWLDPEIPP	1205.620	603.314		•						
**Asn (N) Related**										
pENW	430.172		•	•	•	•	•		•	2MH^+1^ (*m*/*z* 859.337)
pENWP	527.225			•	•	•	•			
pENWPGP	681.299			•						
pENWPGPK	809.394	405.201		•			•			
pENWPSP	711.310				•	•	•			
pENWPSPK	839.405	420.206			•	•	•			known as BPP-7b
pENWPSPKVPP	1132.579	566.793			•	•	•			known as BPP-10e
**Arg (R) Related**										
pERW	472.230		•	•	•	•	•	•		2MH^+1^ (*m*/*z* 859.337)
pERWPGP	723.357		•						•	
pERWPGPEIPP	1159.590	580.299							•	
pERWPGPK	851.453	426.230	•						•	
pERWPGPKVPP	1144.626	572.817	•						•	
pERW_ox_PGPKVPP	1160.621	580.814	•						•	Trp oxidation
pERW_diox_PGPKVPP	1176.616	588.812							•	Trp dioxidation
pERWPGPKVPPL	1257.710	629.359	•						•	
pERWPGPKVPPLE	1386.753	693.881	•							identical to ID: A0A1I9KNP8
**Further Peptides**										
pEKY	421.208		•	•	•	•	•	•	•	
pEDW	431.156			•						
pEDWR	587.258			•						
pELSPR	583.320							•		
pEHPGGGGGGW	892.370	446.688			•	•	•		•	pHpG-related
pERRPPEIPP	1072.590	536.799	•		•	•				
WPGPKVPP	877.493	439.250	•						•	
pEMWPGPKVPP	1119.566	560.287	•							
**Natriuretic Peptide Related**										
DNEPP	571.236			•						
DNEPPKKVPPN	1234.643	617.825	•							
EDNEPP	700.278	350.643							•	
EDNEPPKKLPPS	1350.690	675.849							•	
IGSVSGLGC_CAM_NK	1091.551	546.279		•	•		•	•		BU tryptic digest, protected Cys
IGSHSGLGC_CAM_NK	1129.542	565.275							•	BU tryptic digest, protected Cys

a Tandem MS/MS confirmed sequences of snake venom metalloproteinase inhibitors (svMP-i), BPP and natriuretic peptides (NP) of seven viper venoms. Masses are given in monoisotopic (mono) *m/z* and if observed with double charges (*z* = 2). Black dots mark the present of a peptide in the corresponding venom. Headline amino acid relation based on the modular pEXW, with pE for pyroglutamate and X for the mentioned amino acid. Amino acid I was set in similarities to known sequences, since a MS differentiation between isobaric L and I was not possible. Post-translational modification written out under “Notes”, as well as further information and carbamidomethyl (CAM).

Different BBP and *C*-terminal truncated sequences of variable length, from three to 12 amino acids, have been annotated in each of the viper venoms (Table 1). The shortest, tripeptidic sequences are henceforth referred to as svMP-i. These small peptides are predicted to protect the venom from autodigestion by its own svMP.^137,138^ The three svMP-i (pEKW, pENW, pERW) are highly abundant, with pEKW often as main representative, and were detected in all seven venoms, except pENW, that could not be observed in the *M. xanthina* venom, and pERW in the *D. palaestinae* proteome.

Among the >25 oberserved peptides pEKWPVPGPEIPP was in all three *Montivipera* and the *M. l. obtusa* venom the main BPP-related sequence with Lys in second position and for the Asn-related pENWPSPKVPP (known as BPP-10e) is exclusive for *Montivipera* and pENWPGPK for *V. darevskii*. The Arg-related BPP were only abundant in the venoms of *V. b. barani* and *D. palaestinae* with various truncations of pERWPGPKVPPLE in both and pERWPGPEIPP in *D. palaestinae* only. The 12-mer pERWPGPKVPPLE is identical to a building block of a *V. ammodytes* BPP-NP precursor (ID: A0A1I9KNP8_VIPAA) and a *V. aspis* BBP (ID: P31351). Based on our observation, the BPP in Viperinae venoms following the modular structure of pEXW(PZ)_1–2_P(EI)/(KV)PPLE, with X mainly K/N/R, while other amino acids on position 2 are rare, *Z* = G/S/V and multiple *C*-terminal truncation. Some exclusive sequences, like the pEKWLDPEIPP (*V. darevskii*), pELSPR (*M. l. obtusa*) and pERRPPEIPP (*Vipera* and *Montivipera*), underlines that the whole group of BPP-NP precursor related peptides have a highly variable combination pattern, of which most physiological effects are still unknown. The high similarity to pit viper BPP sequences, suggests similar serious activities on the blood pressure.

The NP are the third group of peptides deriving from the same precursor. They strongly contribute to the lowering of blood pressure by the NP receptors via cGMP-mediated signaling. NP and can be found in various animals as well as the venom of some elapids and vipers.^139^ Their molecular size ranges from 2 to 4 kDa and they are known from highly medical relevant snakes, like taipans (*Oxyuranus*), brown snakes (*Pseudonaja*), kraits (*Bungarus*) and blunt-nosed vipers (*Macrovipera*). In the case of *M. lebetinus* two different NP structures has been described as lebetins: the long lebetin 2 (3943.4 Da, with one disulfide bridge) and the short lebetin 1 (1305.5 Da), which is identical to the lebetin 2 *N*-terminus.^140^ This terminal sequence is known to be important for platelet aggregation inhibition and to prevent collagen-induced thrombocytopenia.^141^ We observed two peptides with sequences similar to the short lebetin 1β (DNKPPKKGPPNG), those are DNEPPKKVPPN in *Vipera* with K2E and G8V, as well as EDNEPPKKLPPS in *Daboia* with an additional *N*-terminal Glu and three substitutions (K2E, G8L and N11S) (Table 1). The longer lebetins were full length detected in the venom of *M. l. obtusa* as expected for a *M. lebetinus* subspecies, but surprisingly also in *M. b. bulgardaghica* with a homologue to lebetin 2α. Further tryptic peptides of NP related sequences, has been observed in *V. darevskii* (gel band 12a), *M. b. bulgardaghica* (16a), *M. xanthina* (10a), *M. l. obtusa* (8a). For example, all genera showed the *C*-terminal IGSVSGLGCNK sequence, with a single amino acid change of H4V, except *Macrovipera*, that had the lebetin 2 identical C-terminal sequence of IGSHSGLGCNK. Therefore, we confirmed the appearance of NP in the venom of all four genera at the proteomics level, which seems to be a constant part of Viperinae venoms in general.

## 5 Summary

Palearctic vipers are a diverse group of venomous snakes with high impact on health and socioeconomic factors that can be found across three continents. By extensive venomics studies on seven taxa from Türkiye within this group, the venom proteome and peptidome was characterized and quantified in detail. Our complementary MS-based workflows revealed high divergence in their abundance of toxin families, following the major, secondary and minor toxin family trend known for Old World vipers. A closer look into the type of toxins and corresponding abundances shows notable variation between the investigated genera of *Vipera*, *Montivipera*, *Macrovipera* and *Daboia*.

Within the genus *Vipera*, *V. b. barani* had a unique venom mostly composed of svSP. This sets it clearly apart from *V. berus* venoms of other localities, but also viperine venoms in general. *V. b. barani* lacks svMP and the peptidome is closer to the highly medical relevant *D. palaestinae* than to the other viper venoms investigated in this study. The venom of *V. darevskii*, is an example of an understudied taxa, which was unknown until now. We could show, that its composition based on different myotoxic and anticoagulant active homologues, as well as an abundant pEKW peptide part of >10% of the total venom composition. Furthermore, within its venom a truncated but presumably self-contained C-terminal CRISP subdomain could be annotated. It includes a ShKT-like, or CRD domain, indicating potential neurological envenoming effects by *V. darevskii*.

We could show important similarities within the genera *Montivipera* and *Macrovipera* on both, proteomics and peptidomics, level. Here, we describe the first genus-wide *Montivipera* venom comparison. The venom compositions across four taxa of the subclades *raddei* and *xanthina* have a consistent appearance, with the Turkish *M. raddei* as an outliner until now. The direct comparison of the three *Montivipera* venom profiles consistently showed a wide range of toxin homologues to highly medical relevant viper species.

The herein investigated venom of *D. palaestinae* is in support of a high venom varation within the genus *Daboia*. As it is known for eastern *Daboia* species to cause locality-based different clinical images after a bite, we could show that also the western taxa have strong compositional differences. The *D. palaestinae* venoms of Türkiye and Israel display different toxin abundances. Therefore, based on our findings it seems reasonable to expect that a high venom diversity like in Indian *D. russelii* might also be therapeutically relevant for *D. palaestinae*, if not even the whole genus *Daboia*.

Beside the well studied toxin families, all here investigated Palearctic viper venoms have a peptide content of at least 9%. They include a spectrum of svMP-i, BPP, pHpG, and NP. We identified the modular consensus sequence pEXW(PZ)_1–2_P(EI)/(KV)PPLE for BPP related peptides in viper venoms. This underscores the intricate nature of snake venom peptidic compounds potentially influencing blood pressure. Notably, they exhibit an increased impact on the venom composition, as evidenced by their prevalence not only in our seven vipers but also across various other viper species. Peptides found to be distributed in high proportions, equal to major toxin families, and, intriguingly, reaching even higher concentrations based on the small molecular weight. This points to the significance of BPP as well as NP in the overall venom composition, highlighting their potential role in the physiological effects following snakebite envenomings, but might be often overlooked until now.

The study of the herein investigated seven Palearctic viper venoms shows, that their venoms include a variety of different potent peptide and toxin families. Since vipers in Türkiye are responsible for numerous hospitalizations of adults as well as children across the country, deciphering these venom variations is of great interest. Our data on the detailed venom compositions and the comparison to other proteomes, will contribute to provide novel biochemically and evolutionary insights in Old World viper venoms and emphasize the potential medical importance of neglected taxa. In particular, the first venom descriptions of several Turkish viper taxa, will facilitate the risk assessment of snakebite envenoming by these vipers and aid in predicting the venoms pathophysiology and clinical treatments.

## Supporting Information Available

Venom pool information of the seven Palearctic viper venoms; database of Palearctic viper proteomes; detailed snake venomics quantification and peptidomics; bottom-up identified tryptic peptide sequences; top-down identified protein sequences; and dimeric disintegrin pairing in *M. l. obtusa* venom (XLSX)
Venom profiles of the seven Palearctic viper venoms (PDF)

## Abbreviations

ABC: ammonium hydrogen carbonate
ACN: acetonitrile
BPP: bradykinin-potentiating peptides
CTL: C-type lectin-related proteins and snake venom C-type lectins
CRISP: cysteine-rich secretory proteins
DAD: diode array detector
DC: disintegrin-like/cysteine-rich proteins
DI: disintegrins
DTT: dithiothreitol
HFo: formic acid
KUN: Kunitz-type inhibitors
LAAO: l-amino acid oxidases
MES: 2-(*N*-morpholino)ethane sulfonic acid
NGF: nerve growth factors
NP: natriuretic peptides
PDE: phosphodiesterases
pE: pyroglutamate
pHpG: poly-His-poly-Gly
PLA_2_: snake venom phospholipases A_2_
SDS: sodium dodecyl sulfate
svMP: snake venom metalloproteinases
svMP-i: snake venom metalloproteinase inhibitors
svSP: snake venom serine proteases
VEGF: vascular endothelial growth factors F
5N: 5′-nucleotidases
