# Supplementary material for: Venomics and Peptidomics of Palearctic Vipers: A Clade-Wide Analysis of Seven Taxa of the Genera Vipera, Montivipera, Macrovipera, and Daboia across Türkiye
Source: J Proteome Res. 2024 Jul 9;23(8):3524–41. doi: 10.1021/acs.jproteome.4c00171 (PMC11301686; doi:10.1021/acs.jproteome.4c00171)
Supplement: Supplementary file 2 — pr4c00171_si_002.pdf [file pr4c00171_si_002.pdf]

Venomics and Peptidomics of Palearctic vipers: Clade-wide analysis of seven taxa of the genera *Vipera, Montivipera, Macrovipera* and *Daboia* across Türkiye

SUPPLEMENTARY MATERIAL

Maik Damm [1,2,3], Mert Karış [4], Daniel Petras [5,6], Ayse Nalbantsoy [7], Bayram Göçmen [8#], Roderich D. Süssmuth [1]**

**Shared Corresponding Authors (maik.damm@agrar.uni-giessen.de; roderich.suessmuth@tu-berlin.de), #deceased*

[1] Institut für Chemie, Technische Universität Berlin, Straße des 17. Juni 135, 10623 Berlin, Germany
[2] LOEWE-Centre for Translational Biodiversity Genomics, Senckenberganlage 25, 60325 Frankfurt Am Main, Germany
[3] Institute for Insect Biotechnology, Justus-Liebig University Giessen, Heinrich-Buff-Ring 26-32, Gießen, 35392 Germany
[4] Program of Laboratory Technology, Department of Chemistry and Chemical Process Technologies, Acıgöl Vocational School of Technical Sciences, Nevşehir Hacı Bektaş Veli University, 50140 Acıgöl, Nevşehir, Türkiye
[5] Department of Biochemistry, University of California Riverside, 169 Aberdeen Dr, Riverside, CA 92507
[6] Interfaculty Institute of Microbiology and Infection Medicine, University of Tuebingen, Auf der Morgenstelle 24, 72076 Tuebingen, Germany
[7] Department of Bioengineering, Faculty of Engineering, Ege University, 35100, Bornova, Izmir, Türkiye
[8] Zoology Section, Department of Biology, Faculty of Science, Ege University, 35100 Bornova, Izmir, Türkiye

KEYWORDS: venom, snakebite, proteomics, peptidomics, viper

Table of Contents

Additional Materials and Methods *S2*
Detailed Bottom-up proteomics - Snake Venomics *S2*
SDS-PAGE profiling and tryptic digestion *S2*
Detailed Bottom-up proteomics - Mass Spectrometry *S3*
Detailed Top-down proteomics - Mass Spectrometry *S3*
Detailed proteome quantification *S4*

Figure S1: Venom profile of *Vipera berus barani*. *S5*
Figure S2: Venom profile of *Vipera darevskii*. *S6*
Figure S3: Venom profile of *Montivipera bulgardaghica bulgardaghica*. *S7*
Figure S4: Venom profile of *Montivipera bulgardaghica albizona*. *S8*
Figure S5: Venom profile of *Montivipera xanthina*. *S9*
Figure S6: Venom profile of *Macrovipera lebetina obtusa*. *S10*
Figure S7: Venom profile of *Daboia palaestinae*. *S11*

Table S1: Venom pool information of the seven Palearctic viper venoms. *
Table S2: Database of Palearctic viper proteomes. *
Table S3: Quantification of the *V. b. barani* venom pool proteome. *
Table S4: Quantification of the *V. darevskii* venom pool proteome. *
Table S5: Quantification of the *M. b. bulgardaghica* venom pool proteome. *
Table S6: Quantification of the *M. b. albizona* venom pool proteome. *
Table S7: Quantification of the *M. xanthina* venom pool proteome. *
Table S8: Quantification of the *M. l. obtusa* venom pool proteome. *
Table S9: Quantification of the *D. palaestinae* venom pool proteome. *
Table S10: Snake Venomics annotation of the *V. b. barani* venom by pFind. *
Table S11: Snake Venomics annotation of the *V. darevskii* venom by pFind. *
Table S12: Snake Venomics annotation of the *M. b. bulgardaghica* venom by pFind. *
Table S13: Snake Venomics annotation of the *M. b. albizona* venom by pFind. *
Table S14: Snake Venomics annotation of the *M- xanthina* venom by pFind. *
Table S15: Snake Venomics annotation of the *M. l. obtusa* venom by pFind. *
Table S16: Snake Venomics annotation of the *D. palaestinae* venom by pFind. *
Table S17: Top-down annotation of the *V. b. barani* venom by TopPIC. *
Table S18: Top-down annotation of the *V. darevskii* venom by TopPIC. *
Table S19: Top-down annotation of the *M. b. bulgardaghica* venom by TopPIC. *
Table S20: Top-down annotation of the *M. b. albizona* venom by TopPIC. *
Table S21: Top-down annotation of the *M. xanthina* venom by TopPIC. *
Table S22: Top-down annotation of the *M. l. obtusa* venom by TopPIC. *
Table S23: Top-down annotation of the *D. palaestinae* venom by TopPIC. *
Table S24: Dimeric disintegrins in the *M. l. obtusa* venom. *
**Detailed supplementary tables are in the corresponding xlsx file.*

Additional Materials and Methods

Detailed Bottom-up proteomics - Snake Venomics:

Venom fractionation

For the analysis of each venom pool, 1 mg of lyophilized venom was dissolved to a final concentration of 10 mg/mL in aqueous 5% (*v*/*v*) acetonitrile (ACN) with 1% (*v*/*v*) formic acid (HFo) and centrifuged for 5 min at 10,000 × g. The supernatants were fractionated on a reversed-phase Supelco Discovery BIO wide Pore C18-3 (4.6 × 150 mm, 3 µm particle size) column operated by a HPLC Agilent 1200 (Agilent Technologies, Waldbronn, Germany) chromatography system. The following gradient with ultrapure water with 0.1% (v/v) HFo (solvent A) and ACN with 0.1% (v/v) HFo (solvent B) was used at 1 mL/min, with a linear gradient between the time points, given at min (B%): 0–5 (5% const.), 5–100 (5 to 40%), 100–120 (40 to 70%), 120–130 (70% const.), and 5 min re-equilibration at 5% B. The chromatography runs were observed by a diode array detector (DAD) at λ = 214 nm detection wavelength. Samples were collected through time-based fractionation (1 fraction/min) and combined peak fractions were dried in a centrifugal vacuum evaporator.

Peaks later than 25 min were further processed by the snake venomics steps of gel separation and tryptic digest, peaks with earlier retention times (R_t) are known for their low molecular mass peptide content and were directly sent to the LC-MS. The viperine abundant tripeptide pEKW (with pE for pyroglutamate) signal at around 25 min was set as benchmark.

SDS-PAGE profiling and tryptic digestion

The dried venom fractions were redissolved in 10 µL reducing 2× sodium dodecyl sulfate (SDS) sample buffer (125 mM Tris HCl pH 6.8, 4% (w/v) SDS, 17.5% (w/v) glycerol, 0.02% (w/v) Bromphenol blue and 200 mM freshly prepared dithiothreitol DTT in ultra-pure (MQ) water), heated for 10 min at 95 °C, fully loaded and separated using a 12% SDS-PAGE (SurePage Bis-Tris, Genscript, Piscataway, NJ, USA) run with MES buffer (50 mM 2-(*N*-morpholino)ethane sulfonic acid (MES), 50 mM Tris base, 1 mM EDTA, 0.1% (w/v) SDS, stored in brown glass flasks at 4°C) at 200 V for 21 min. A PageRuler Unstained Protein Ladder (Thermo Scientific, Waltham, MA, USA) was used as protein mass standard. Gels were three times short-washed with water. Proteins were fixed with preheated (50-60 °C) fixation buffer three times for 10 min each (aqueous, 40% (v/v) methanol, 10% (v/v) acetic acid), stained for 45 min in preheated (50-60 °C) fast staining buffer (aqueous, 0.3% (v/v) HCl 37%, 100 mg/L Coomassie 250G) under constant mild shaking, and kept overnight at 4 °C in storage buffer (aqueous, 20% (v/v) methanol, 10% (v/v) acetic acid) for destaining. The cleaned gels were then scanned for documentation and quantification. Gel pieces with single protein bands were cut, dried with 500 µL ACN, and stored at −20 °C without ACN until tryptic digestion. The disulfide bridges were reduced with 30 µL freshly prepared DTT (100 mM in 100 mM ammonium hydrogen carbonate (ABC) per gel band) for 30 min at 56 °C and dried with 500 µL ACN for 10 min before removing the supernatant. Cysteines were alkylated with freshly prepared iodoacetamide (55 mM in 100 mM ABC) for 20 min at room temperature in the dark to protect the reduced thiols from oxidation and washed with 500 µL ACN for 2 min. before removing the supernatant. Gel samples were dried again with 500 µL ACN for 15 min, ACN removed, followed by 30 min incubation on ice with 30 µL freshly activated trypsin (13.3 ng/µL, 10% (v/v) ACN in 10 mM ABC; Pierce trypsin, Thermo, Rockfeld, IL, USA). When necessary, additional volumes of trypsin were added, so that the gel piece was still covered in buffer. All samples were incubated for 90 min on ice, 20 µL ABC buffer (10 mM) was added, and were incubated overnight at 37 °C. Peptides were extracted with 100 µL pre-warmed elution buffer (aqueous, 30% (v/v) ACN MS grade, 5% (v/v) HFo) at 37 °C for 30 min. The supernatant was transferred into a separate microtube, vacuum-dried and if possible directly prepared for the LC-MS/MS measurement, else samples were stored at −20 °C.

Detailed Bottom-up proteomics - Mass Spectrometry:

The following gradient with ultrapure water with 0.1% (*v*/*v*) HFo (solvent A) and ACN with 0.1% (*v*/*v*) HFo (solvent B) was used at 0.3 mL/min, with a linear gradient between the time points, given at min (B%): 0–1 (5% const.), 1–11 (5 to 40%), 11–12 (40 to 99%), 12–13 (99% const.), and 2 min re-equilibration at 5% B. The parameters in the ESI positive modus were as follows: 270 °C capillary temperature, 45 L/min sheath gas, 10 L/min auxiliary gas, 4.0 kV source voltage, 100.0 µA source current, 20 V capillary voltage, 130 V tube lens. FTMS measurements were performed with 1 scans and 1000 ms maximal fill time. AGC targets were set to 10^6 for full scans and to 3×10^5 for MS2 scans. MS2 scans were performed with a mass resolution (R) of 60,000 (at *m*/*z* 400) for *m*/*z* 250–2000. MS2 spectra were obtained in data-dependent acquisition (DDA) mode as top2 with 35 V normalized CID energy, and 500 as the minimal signal required with an isolation width of 3.0. The default charge state was set to z = 2, and the activation time to 30 ms. Unassigned charge states and charge state 1 were rejected for tryptic digest peptides, for direct submitted fractions from the initial HPLC run all charge states were measured.

Detailed Top-down proteomics - Mass Spectrometry:

For the denaturing TD analysis, 100 µg of lyophilized venom was dissolved to a final concentration of 10 mg/mL in aqueous 1% (*v*/*v*) HFo and centrifuged for 5 min at 20,000 × g. The supernatant was mixed with 30 L of citrate buffer (0.1 M, pH 3.0) and split into two aliquots. The first aliquot was mixed 10 L of 0.5 M tris(2-carboxyethyl)phosphine (TCEP), for reduction of disulfide bonds, and incubated for 30 min at 65 °C. The second was supplemented with 10 L of ultrapure water and will be referred as non-reduced sample. Both samples were centrifuged for 5 min at 20,000 × g and 10 µL of each was injected into an Q Exactive HF mass spectrometer (Thermo, Bremen, Germany) via a Vanquish ultra-high performance liquid chromatography (UHPLC) system (Agilent Technologies, Waldbronn, Germany) using a reversed-phase Supelco Discovery BIO wide C18 (2.0 × 150 mm; 3 m particle size; 300 Å pore size) column thermostated at 30 °C. The following gradient with ultrapure water with 0.1% (*v*/*v*) HFo (solvent A) and ACN with 0.1% (*v*/*v*) HFo (solvent B) was used at 0.4 mL/min, with a linear gradient between the time points, given at min (B%): 0–6 (5% const.), 6–25 (5 to 40%), 25–30 (40 to 70%), 30–35 (70% const.), and 5 min re-equilibration at 5% B. The parameters in the ESI positive modus were as follows: 265.50 °C capillary temperature, 50.00 AU sheath gas, 12.50 L/min auxiliary gas, 3.50 kV source voltage, 100.00 µA source current. FTMS measurements were performed with 1 scans and 1000 ms maximal fill time. MS2 scans were performed with a mass resolution (R) of 140,000 (at *m*/*z* 200). MS2 spectra were obtained in DDA mode as top3 with 30% normalized high energy C-trap dissociation (HCD) and an isolation window of *m*/*z* 3.0. The default charge state was set to z = 6, and the activation time to 30 ms. Unassigned charge states and isotope states were rejected for MS2 measurements.

Detailed proteome quantification:

The comparable approach determine a toxin family abundance in the venom as the sum of all its normalized toxin abundances *T*:

$$T_{toxin\ family} = \sum T_{band}$$
$$\text{with } T_{all\ families} = 1$$

The normalized toxin abundance within a single protein band T_{band} is calculated with the normalized values of the RP-HPLC peak integral *P* measured at 214 nm, the densitometric gel band intensity *D* and if necessary the relative MS ion intensity *M* of the most abundant and identified peptides:

$$T_{band} = P \cdot D \cdot M$$

For the peak quantification after blank run subtraction, the HPLC separation chromatogram fractions were integrated as area under the curve P_{peak} in ratio to the total sum of all peaks:

$$P = \frac{P_{peak}}{\sum P_{all\ HPLC\ peaks}}$$

For the densitometric quantification of a single SDS band, the non-highly compressed gel scan (here in PNG format) was processed by Fiji [82]. The colour depth was set to 8bit grayscale and inverted to integrate former darker bands with higher values. The band area A_{band} and the corresponding integrated band densities D_{band} were measured for each band, as well as a corresponding background areas A_{bg} and integrated band densities D_{bg}. By removing the proportion of background, we calculated the normalized gel band intensity *D* for each toxin band in the gel:

$$D = \frac{D_{band-bg}}{\sum D_{band-bg}} = \frac{D_{band} - (A_{band} \cdot \frac{D_{bg}}{A_{bg}})}{\sum D_{band-bg}}$$

In case of multiple toxin identification within a single band, single normalized toxin abundances *M* were estimated based on the ion intensity sum of the three most intensive peptide ions of one toxin from M_3 in relation to the sum of all top3 toxin ions from the other co-migrated toxins families within this MS sample, as summarised in Calvete *et al.* 2023:

$$M = \frac{M_{3\ of\ toxin\ family\ in\ band}}{\sum M_{3\ of\ all\ toxin\ families\ in\ band}}$$

In total, band identification based on the BU, TD and peptidomics results, in comparison to the IMP and the apparent masses of the SDS bands.

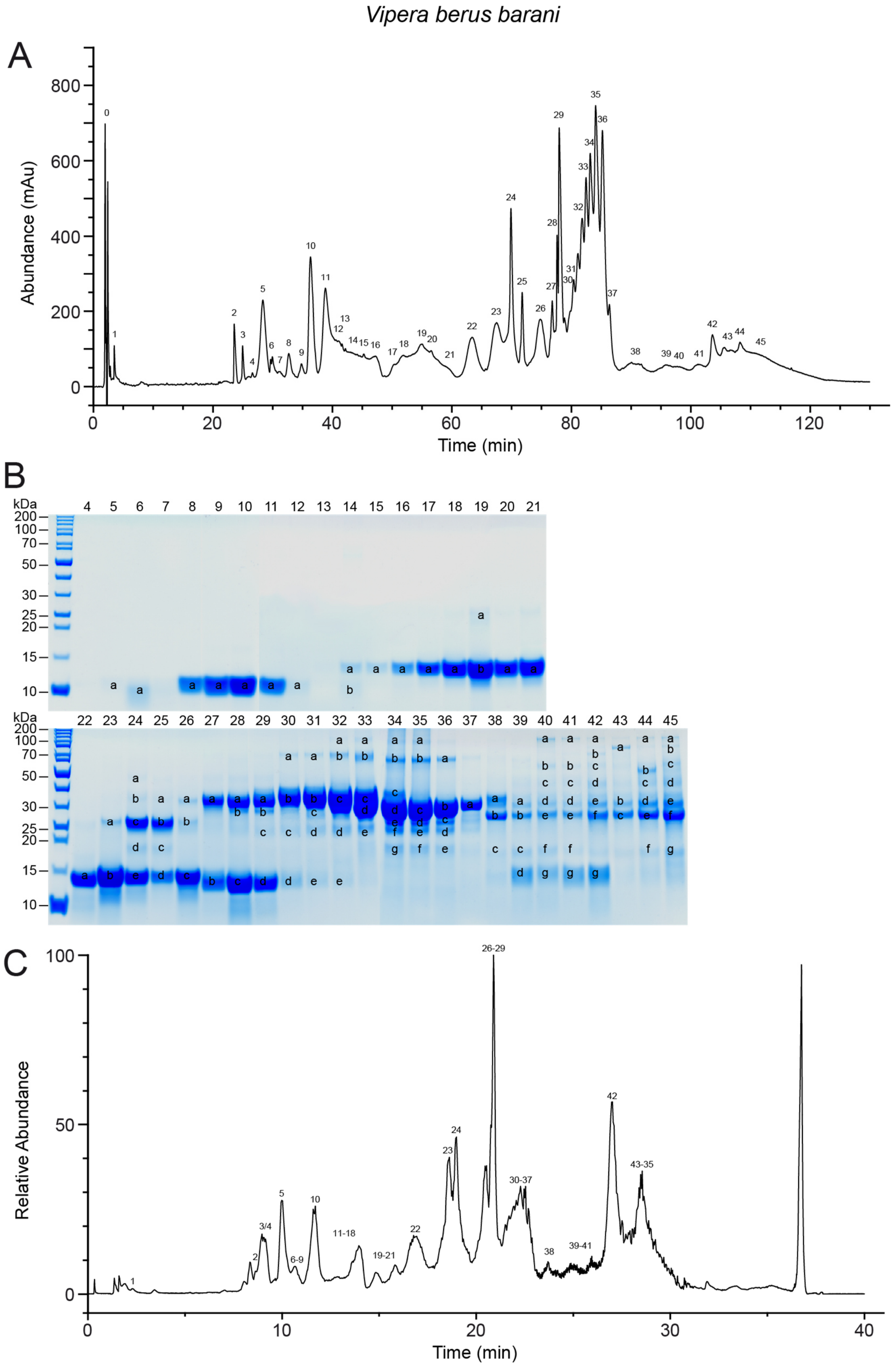

Supplementary Figure S1: Venom profile of *Vipera berus barani*.
For the bottom-up snake venomics approach the **(A)** RP-HPLC profile was observed at λ = 214 nm, with peak 0 corresponding to the injection peak, and venom fractions further analyzed by **(B)** Coomassie-stained SDS-PAGE under reducing conditions. PAGE line nomenclature is based on RP-HPLC fractions from (A). Labelled bands were cut, subjected to tryptic digestion, and analyzed with LC-MS. For top-down and intact mass profiling the venom profile was observed via the **(C)** total ion current chromatogram by ESI under non-reducing conditions.

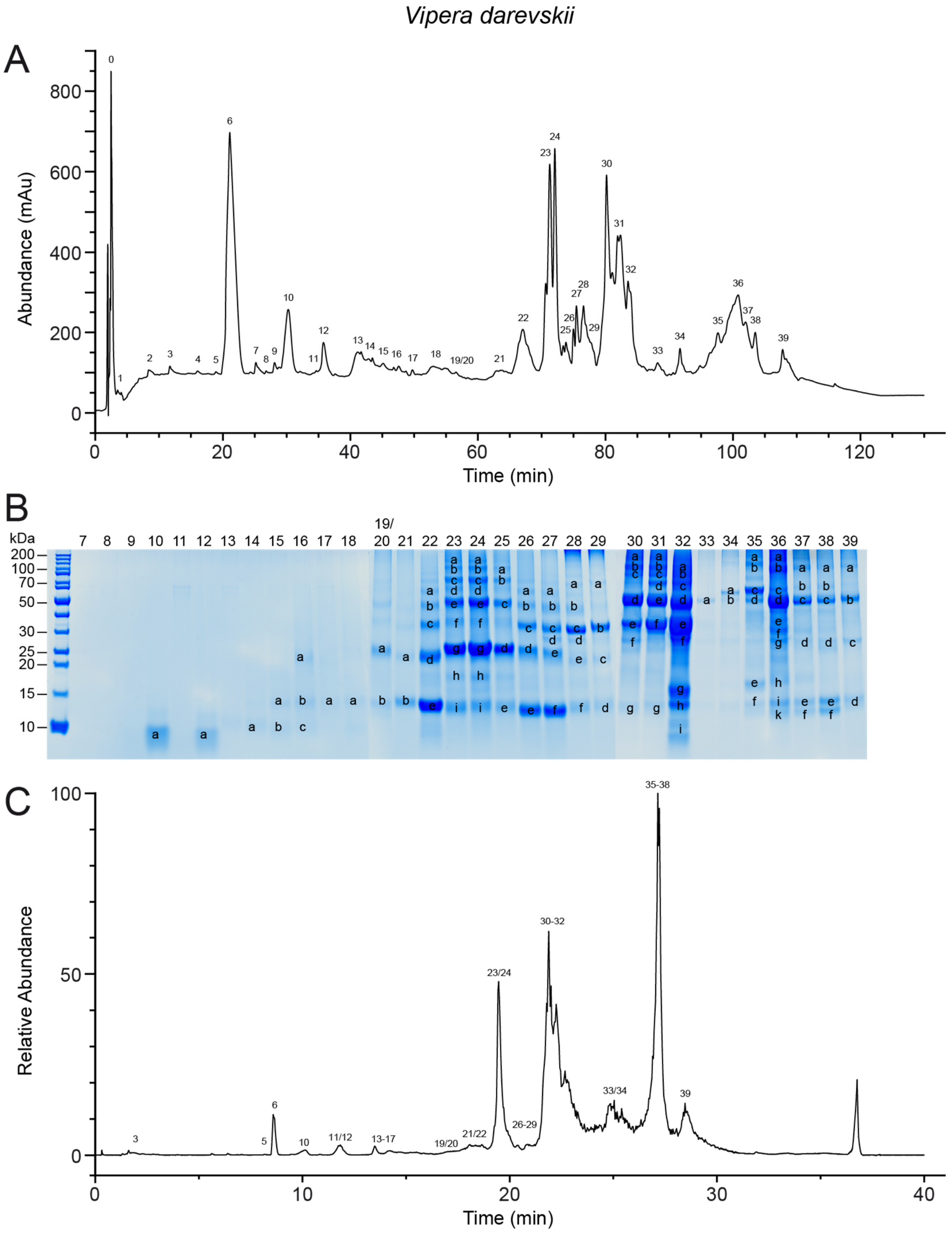

Supplementary Figure S2: Venom profile of *Vipera darevskii*.
For the bottom-up snake venomics approach the **(A)** RP-HPLC profile was observed at λ = 214 nm, with peak 0 corresponding to the injection peak, and venom fractions further analyzed by **(B)** Coomassie-stained SDS-PAGE under reducing conditions. PAGE line nomenclature is based on RP-HPLC fractions from (A). Labelled bands were cut, subjected to tryptic digestion, and analyzed with LC-MS. For top-down and intact mass profiling the venom profile was observed via the **(C)** total ion current chromatogram by ESI under non-reducing conditions.

Montivipera bulgardaghica bulgardaghica

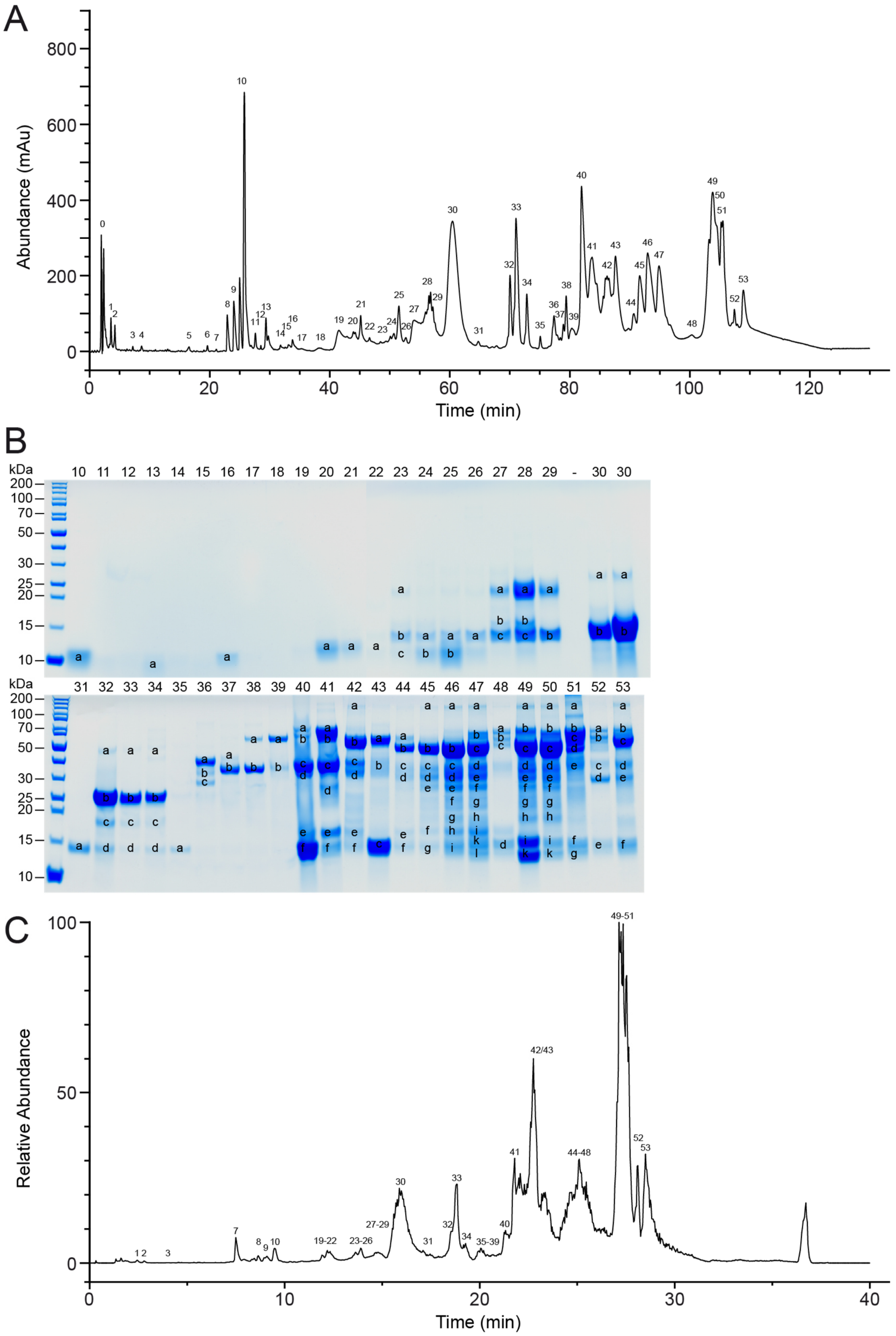

Supplementary Figure S3: Venom profile of *Montivipera bulgardaghica bulgardaghica*.
For the bottom-up snake venomics approach the **(A)** RP-HPLC profile was observed at λ = 214 nm, with peak 0 corresponding to the injection peak, and venom fractions further analyzed by **(B)** Coomassie-stained SDS-PAGE under reducing conditions. PAGE line nomenclature is based on RP-HPLC fractions from (A). Labelled bands were cut, subjected to tryptic digestion, and analyzed with LC-MS. For top-down and intact mass profiling the venom profile was observed via the **(C)** total ion current chromatogram by ESI under non-reducing conditions.

Montivipera bulgardaghica albizona

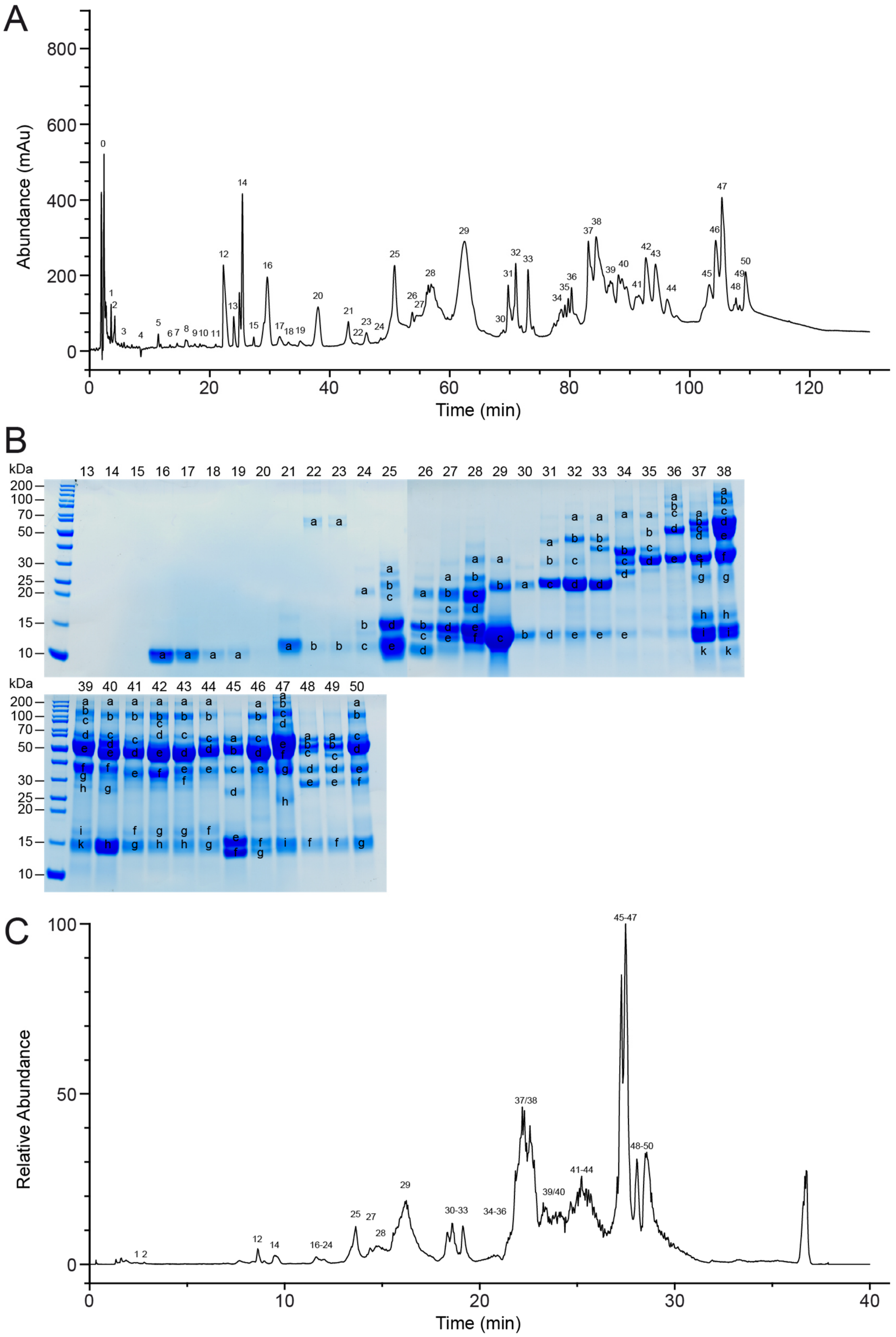

Supplementary Figure S4: Venom profile of *Montivipera bulgardaghica albizona*.
For the bottom-up snake venomics approach the **(A)** RP-HPLC profile was observed at λ = 214 nm, with peak 0 corresponding to the injection peak, and venom fractions further analyzed by **(B)** Coomassie-stained SDS-PAGE under reducing conditions. PAGE line nomenclature is based on RP-HPLC fractions from (A). Labelled bands were cut, subjected to tryptic digestion, and analyzed with LC-MS. For top-down and intact mass profiling the venom profile was observed via the **(C)** total ion current chromatogram by ESI under non-reducing conditions.

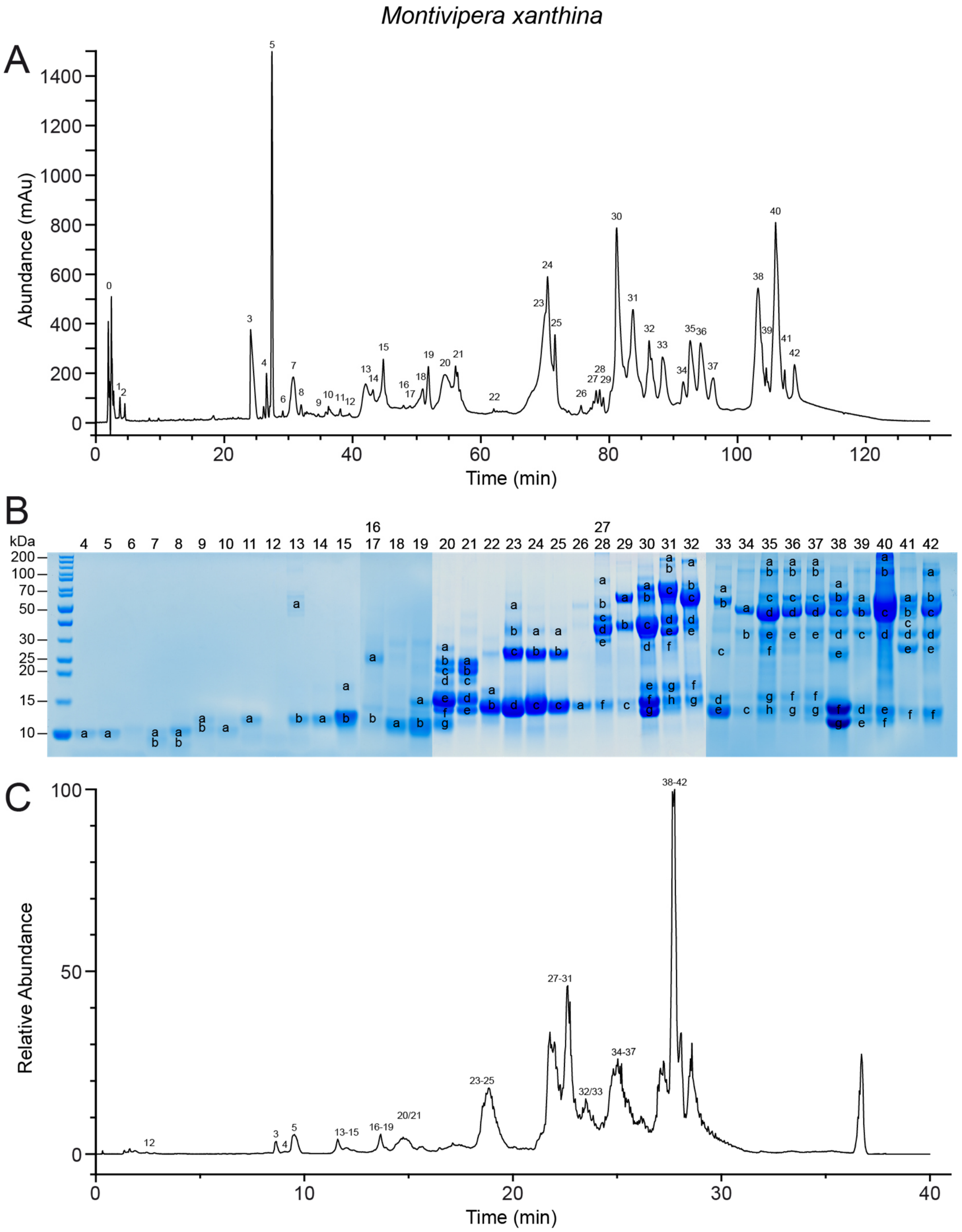

Supplementary Figure S5: Venom profile of *Montivipera xanthina*.
For the bottom-up snake venomics approach the **(A)** RP-HPLC profile was observed at λ = 214 nm, with peak 0 corresponding to the injection peak, and venom fractions further analyzed by **(B)** Coomassie-stained SDS-PAGE under reducing conditions. PAGE line nomenclature is based on RP-HPLC fractions from (A). Labelled bands were cut, subjected to tryptic digestion, and analyzed with LC-MS. For top-down and intact mass profiling the venom profile was observed via the **(C)** total ion current chromatogram by ESI under non-reducing conditions.

Macrovipera lebetinus obtusa

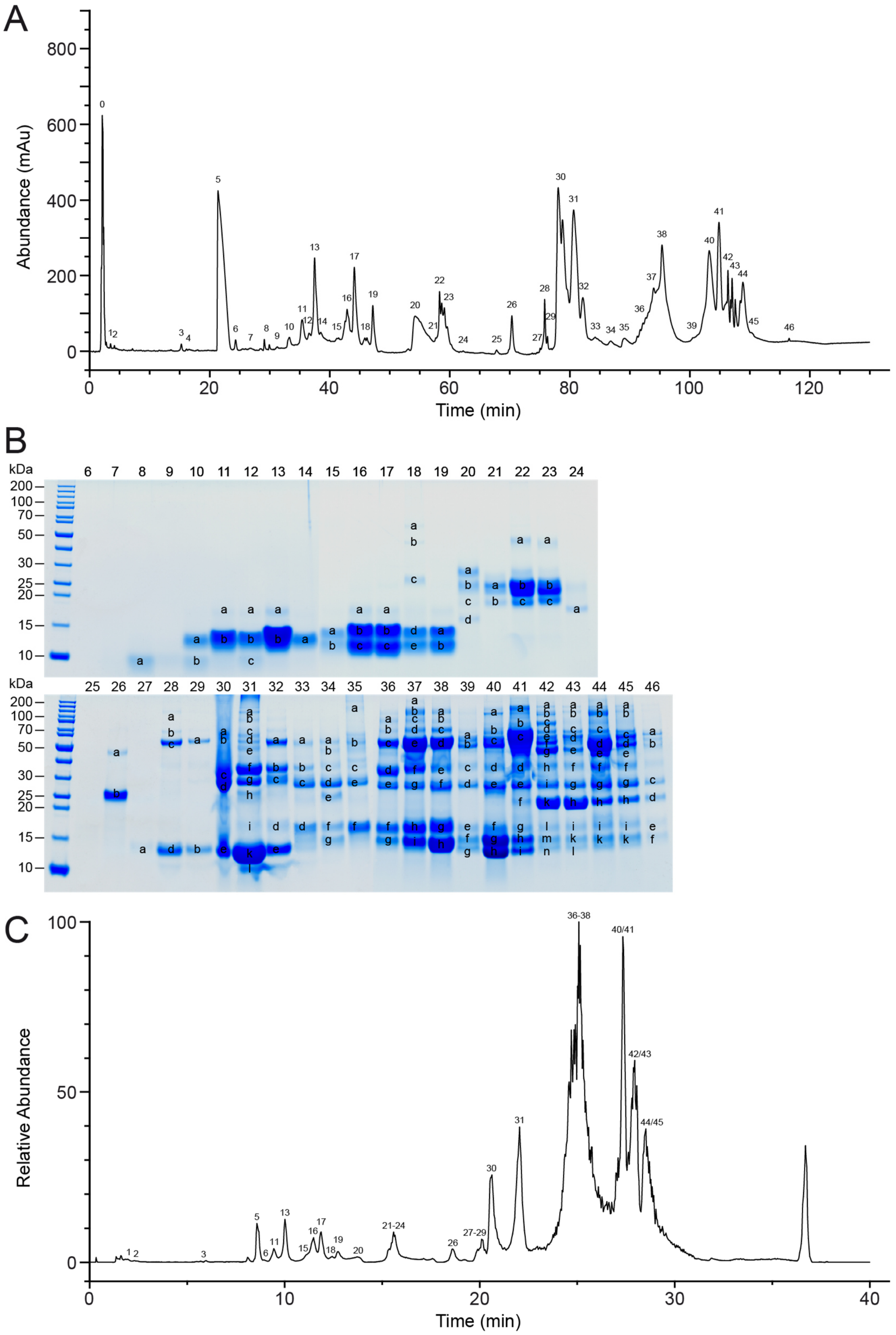

Supplementary Figure S6: Venom profile of *Macrovipera lebetina obtusa*.
For the bottom-up snake venomics approach the **(A)** RP-HPLC profile was observed at λ = 214 nm, with peak 0 corresponding to the injection peak, and venom fractions further analyzed by **(B)** Coomassie-stained SDS-PAGE under reducing conditions. PAGE line nomenclature is based on RP-HPLC fractions from (A). Labelled bands were cut, subjected to tryptic digestion, and analyzed with LC-MS. For top-down and intact mass profiling the venom profile was observed via the **(C)** total ion current chromatogram by ESI under non-reducing conditions.

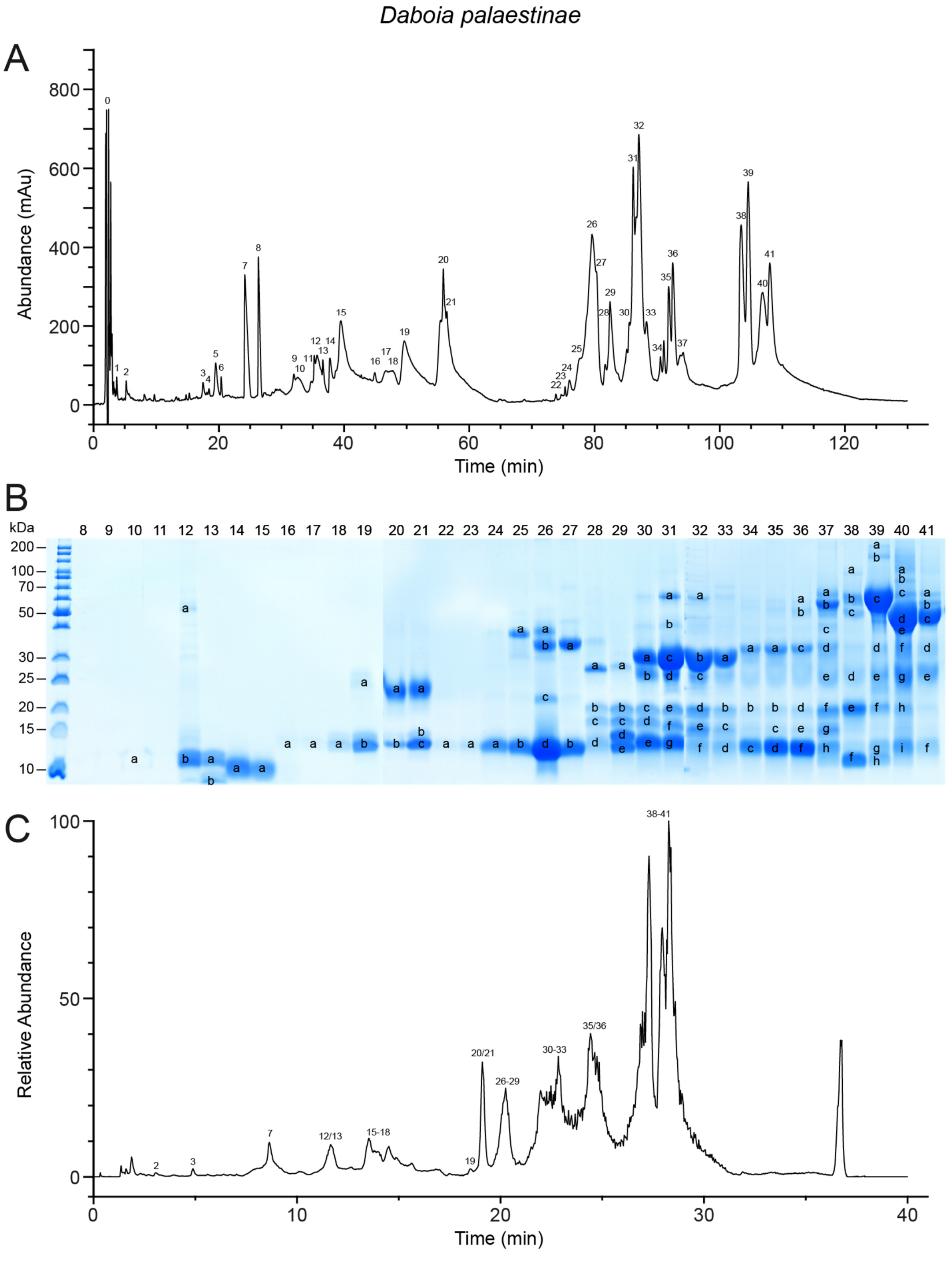

Supplementary Figure S7: Venom profile of *Daboia palaestinae*.
For the bottom-up snake venomics approach the **(A)** RP-HPLC profile was observed at λ = 214 nm, with peak 0 corresponding to the injection peak, and venom fractions further analyzed by **(B)** Coomassie-stained SDS-PAGE under reducing conditions. PAGE line nomenclature is based on RP-HPLC fractions from (A). Labelled bands were cut, subjected to tryptic digestion, and analyzed with LC-MS. For top-down and intact mass profiling the venom profile was observed via the **(C)** total ion current chromatogram by ESI under non-reducing conditions.
